# Associations between Prenatal Exposure to Phthalates and Timing of Menarche and Growth and Adiposity into Adulthood: A Twenty-Years Birth Cohort Study

**DOI:** 10.3390/ijerph18094725

**Published:** 2021-04-29

**Authors:** Ye’elah E. Berman, Dorota A. Doherty, Katharina M. Main, Hanne Frederiksen, Martha Hickey, Jeffrey A. Keelan, John P. Newnham, Roger J. Hart

**Affiliations:** 1 Women and Infants Research Foundation, King Edward Memorial Hospital, Perth 6008, Australia; yeelah.berman@uwa.edu.au (Y.E.B.); dorota.doherty@uwa.edu.au (D.A.D.); jeff.keelan@uwa.edu.au (J.A.K.); john.newnham@uwa.edu.au (J.P.N.); 2 Division of Obstetrics and Gynaecology, University of Western Australia, Perth 6008, Australia; 3 Department of Growth and Reproduction, International Centre for Research and Research Training in Endocrine Disruption of Male Reproduction and Child Health (EDMaRC), University of Copenhagen-Rigshospitalet, Blegdamsvej 9, DK-2100 Copenhagen, Denmark; Katharina.Main@regionh.dk (K.M.M.); Hanne.Frederiksen@regionh.dk (H.F.); 4 Department of Obstetrics and Gynaecology, The University of Melbourne, Melbourne 3010, Australia; hickeym@unimelb.edu.au

**Keywords:** growth, age at menarche, adiposity, phthalate metabolites, girls, antenatal exposure

## Abstract

Phthalates are ubiquitous environmental chemicals with endocrine disrupting properties and potentially obesogenic effects. We hypothesised that antenatal phthalate exposure may influence growth and adiposity patterns in girls through childhood into adolescence. Among 1342 Raine Study singleton females, 462 had maternal serum and at least one outcome available up to 20 years of age. Individuals’ maternal serum collected at 18 and 34 weeks gestation was pooled and analyzed for concentrations of 32 metabolites of 15 phthalate diesters. Cox regression and linear models were used to determine associations between maternal phthalate levels and age at menarche, change in height and weight z-scores between birth and two years, height from birth to 20 years, BMI from two to 20 years, deviation from mid-parental height at age 20 and DEXA scan measures at age 20. Weak negative associations were detected with some phthalate metabolites and change in height and weight z-score during infancy. Weak positive associations between some of the high molecular weight phthalate metabolites and height z-score were detected during childhood. While still within the normal range, age at menarche was slightly delayed in girls with higher prenatal exposure to the higher molecular weight phthalate metabolites. We derived some associations between prenatal phthalate exposure with early growth patterns and age at menarche.

## 1. Introduction

It is increasingly acknowledged that environmental factors may adversely affect human health. Strong evidence exists from animal models that exposure to endocrine disrupting chemicals can exert adverse effects in the offspring, ranging from influences on behaviour, metabolic risk, growth and reproductive development and function [1,2]. One group of endocrine disrupting chemicals, the diesters of phthalic acid (phthalates), are ubiquitous within our environment, and phthalate metabolites are detectable within the majority of the population at any one time [3,4]. They are widely used in industrial and consumer products such as various plastics and personal care products. Exposure occurs through ingestion, dermal absorption [5], inhalation [6] (http://www.atsdr.cdc.gov, accessed on 20 August 2020) and across the placenta [7,8]. Once absorbed, phthalates are rapidly hydrolysed to monoesters and some of the more nonpolar metabolites are further oxidized and/or glucuronidated [9]. Any potential endocrine disrupting influence from their exposure is often complex in nature, as effects may be confounded by additive influences from other endocrine disrupting chemicals [10], and phthalate metabolite actions may not follow standard linear dose response curves.

In a cohort of Western Australian adolescent boys, we previously confirmed the continual generational height increase of boys over their parents’ height. We also derived a potential positive association of prenatal exposure to the lower molecular weight phthalate metabolites with growth (measured as height and body mass index (BMI)) through childhood and adolescence [11]. It is well established that over the last century children have been getting taller, including in countries where the average height is generally tall [12] and where overweight and obesity are increasing [13]. Globally, there has been a three-fold increase in the prevalence of obesity over the last four decades [14], although there are signs that childhood obesity may have plateaued in developed countries over recent years [15]. Understanding the early life origins of childhood overweight and obesity are particularly important since they are strong predictors of adverse health outcomes such as the metabolic syndrome [16]. The timing of the growth spurt can predict the onset of metabolic syndrome [17], as well as changes in pubertal timing, which can influence final adult height [18].

Additionally, in this same cohort of Western Australian (Gen 2) girls, we previously reported a trend towards earlier age at menarche in girls exposed to higher levels of the metabolites of di-(2-ethylhexyl) phthalate (DEHP) [19]. With recent advances in phthalate metabolite analysis allowing inclusion of a greater number of metabolites [20], the aim of this study was to evaluate the potential influences of maternal phthalate exposure on their daughter’s age at menarche and growth and body composition through childhood and adolescence. There is a well-established interplay between girls’ growth, adiposity and pubertal maturation [18,21], and potential influences of maternal phthalate exposure on pubertal trajectory. For instance, in a Mexican study, a doubling of gestational mono-benzyl phthalate (MBzP) concentrations was associated with increased odds of being at a higher Tanner stage for breast development at eight to 14 years [22], and there was an association of higher mono-ethyl phthalate exposure with an earlier menarche [23].

Phthalate metabolites have been purported as obesogenic, as they may influence the differentiation of adipocytes [2,24], lipid accumulation and stimulate peroxisome z-activated receptors (PPARs) [25]. Several longitudinal studies have analysed prenatal exposures to phthalate metabolites with respect to subsequent adiposity and height trajectories, with mixed findings [26,27,28,29,30,31]. In the literature the most consistent finding relates to a positive effect on BMI with di-ethyl phthalate (DEP) exposure [27,29,30]. Unlike the potential influence of phthalates on weight gain and adiposity from birth into childhood, there is a paucity of data with respect to postnatal height and development into childhood and adolescence [32].

As evidence suggests that obesity is programmed in-utero [24,33,34], we hypothesised that prenatal exposure to greater concentrations of phthalates would lead to an increase in the trajectories of adiposity as measured by anthropometry and dual energy X-ray absorptiometry (DEXA) through childhood and adolescence. Consequently, due to the interplay of obesity and menarche [21,35], this would result in earlier menarche. Hence, we set out to study potential influences of maternal phthalate exposure on female height, fat accumulation and age at menarche using a well-established birth cohort, the Raine Study.

## 2. Materials and Methods

The materials and methods used in the current paper closely follow those used in the paper examining the effect of maternal serum phthalate levels on growth in boys [11]. As in the previous study, data were sourced from the Raine Study (www.rainestudy.org.au) (accessed on 20 March 2020), which examined associations between early life events and subsequent behaviour and health [36]. Raine Study Gen2 (Gen2) is a cross-section of the larger longitudinal and multigenerational study that investigated the safety and effects of ultrasound on the developing fetus. Between 1989 and 1991, the study recruited 2900 women at 16 to 20 weeks gestation in Western Australia (WA), who delivered 2868 live born children [36]. Detailed anthropometric, respiratory, neurocognitive, endocrine, cardiovascular, physical fitness, metabolic, behavioural, psychological and social measurements were collected via surveys at ages 1, 2, 3, 5, 8, 10, 14, 17 and 20 years. Age at menarche, defined as the age at the onset of the first menstrual period, was prospectively recorded using a purpose-designed questionnaire [37] at ages 8, 10, 14 and 17. If menarche had been reached since the previous follow-up, caregivers were asked to contemporaneously report the exact date of onset. Participants’ heights at each follow-up were measured to the nearest 0.1 cm using a Harpenden Neonatometer (Holtain Ltd., Crosswell, UK) or a Holtain stadiometer (Holtain Ltd.). Weight was measured to the nearest 100 g using hospital scales at birth, and Wedderburn Chair Scales (Wedderburn, Australia), or automatic electric scales thereafter. All measurements at all ages were performed by trained research personnel. Information on parental height, prepregnancy BMI and smoking were collected prospectively. Mid-parental height, a measure of offspring growth potential, was calculated as the average of the mother and father’s height z-score. The New Intrauterine Growth Curves based on United States data [38] were used to calculate participants’ z-score for length and weight at birth. Z-scores for BMI, weight and height for all subsequent surveys were calculated using the Centers for Disease Control and Prevention (CDC) clinical growth charts [39]. Lean mass, soft tissue mass and fat mass were measured by DEXA scan at Gen2–20 year follow-up using the Norland XR-36 densitometer (Norland Medical Systems, Inc., Fort Atkinson, WA, USA), as reported previously [40]. Cohort members could participate in some or all the follow-up assessments.

### 2.1. Study Population

**Figure 1:**
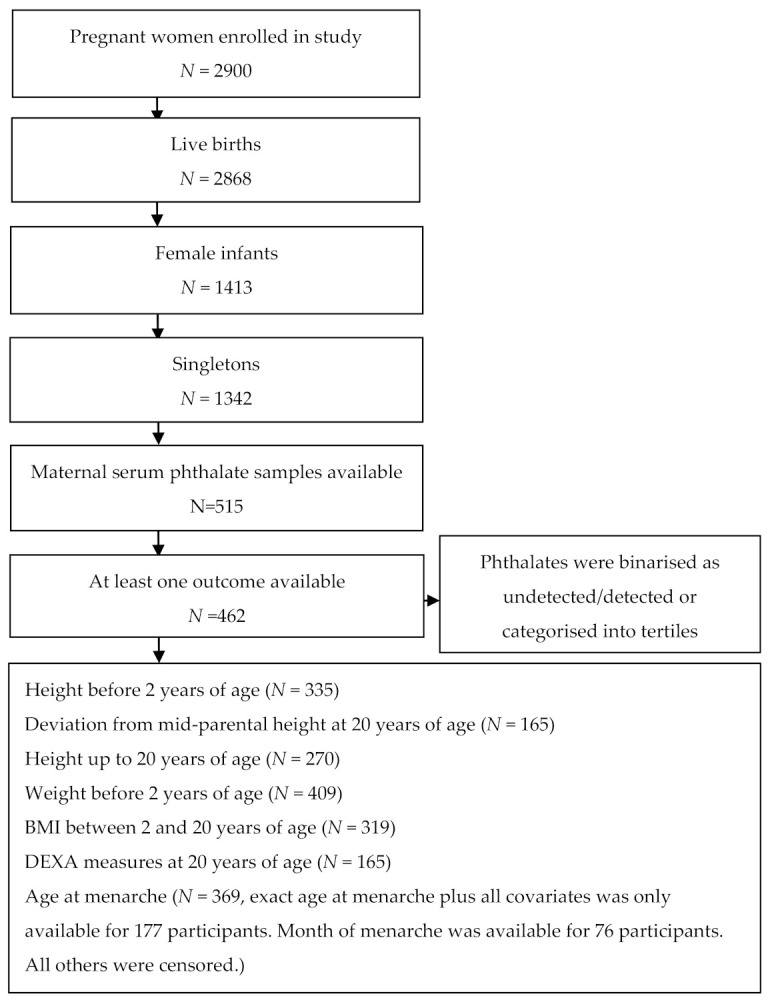
Flow chart of study participants.

The Raine Study is one of the largest and most closely followed prospective cohorts of pregnancy, childhood and adolescence in the world [41]. The study population were adolescent girls from the 2868 live born Raine Study Gen2. Due to the differences in growth patterns between sexes, analyses were performed on girls only. After excluding male infants and multiple births, the resulting population was 1342 singleton females. Maternal phthalate measurements were available for 982 Raine Study infants, and 515 of these were girls. Of these girls, 462 had at least one height, BMI, DEXA or age at menarche outcome available between birth and 20 years of age and were therefore eligible for analysis. The sample size used in each analysis and the selection of the study population are shown in Figure 1. The number of BMI and height measurements used in the linear mixed models at each follow-up are shown in Supplementary Table S1.

### 2.2. Management of Stored Maternal Blood Samples

Serum was collected from maternal blood at 18 and between 34 and 36 weeks gestation, and stored in aliquots without thawing at −80 °C. For each woman, 200 µL aliquots of the 18 and 34 weeks samples were pooled, frozen and couriered to Copenhagen, Denmark. Pooling provided an approximation of antenatal phthalate exposure across gestation. A previous pilot study confirmed stability of samples during prolonged storage at either −80 °C or up to 15 weeks at −20 °C and demonstrated unaltered recovery when processed without acid addition before storing or after storage [19].

### 2.3. Phthalate Measurements

As previously published, maternal serum samples were analyzed by isotope diluted LC-MS/MS with preceding enzymatic deconjugation [4]. Additionally, the method for preparation of serum samples, standard solutions and quality controls was published previously, as well as the instrumental analysis and general method validation of metabolites from 15 different phthalate diesters [20].

### 2.4. Categorisation of Phthalates

Twelve phthalate metabolites of eight phthalate di-esters had detectable levels recorded in at least 10% of samples. Sums of metabolites in nanograms/milliliter were created by summing the molar concentrations of specific metabolites and multiplying by their respective phthalate diester concentrations, as previously published [11,20]. These metabolites and sums of the metabolites and their abbreviation are listed in Table 1. Categorization of maternal serum phthalate metabolite levels was based on data from all women who were pregnant with a female fetus, where the child had any of the outcome measures under evaluation available (*n* = 462). Statistical analyses were not performed for phthalate metabolites where less than 10% of the sample (*n* = 46) were detectable. Where more than one third of the sample (*n* > 154) had undetectable levels, phthalate metabolites were classified into a binary variable (detectable/undetectable). Where fewer than one third of the sample (*n* < 154) had undetectable phthalate metabolite levels, phthalate metabolites were classified into tertiles. Sums of phthalates were also classified into tertiles. Supplementary Table S2 shows the minimum, median, maximum and percent of the samples above the limit of detection (% > LOD) for each sub-analysis.

### 2.5. Statistical Analysis

The primary outcomes were height and weight z-score change from 0 to 2 years, height and BMI z-score from 0 to 20 years, deviation from midparental height z-score at 20 years, DEXA scan measurements at 20 years [total fat mass (g), total lean mass (g), and soft tissue mass (g)] and age at menarche. Categorical data were summarized using frequency distributions, and chi-square tests were used to compare the distribution of outcomes between the girls included (*N* = 462) and excluded (*N* = 880) from the analysis. Continuous data were summarized using medians and inter-quartile ranges, and Mann-Whitney tests were performed to compare group differences. Unadjusted and adjusted multivariable Cox proportional hazard regression modelling was performed to evaluate associations between phthalates and age at menarche. For cases where no menarche data were available, age at menarche was censored at the later available age of the eight or 10-year follow up. If only year of menarche was available (*n* = 12), age at menarche was censored at the age at the start of the year. If only month of menarche was available, age at menarche was taken as an event in the middle of the month (*n* = 76). Where the exact date of menarche was available, age at menarche was taken as an event on that date (*n* = 177).

As in the previous study [11], relationships between categorized phthalate metabolite levels and growth (height or BMI z-score) up to 20 years of age were assessed using linear mixed models. The effect of maternal serum phthalate metabolite levels on growth in each age group of infancy, childhood and adolescence, was assessed by the inclusion of an interaction term between phthalate metabolite level and age group (0 to 2, 2 to 10, 10 to 20). As BMI is not an appropriately sensitive outcome measure in infancy, the 0 to 2 age group was excluded from the BMI model. Participants who had all covariates available, as well as at least one measurement available in each age group, were included in the analysis.

Associations between maternal serum phthalate metabolite level and change in height and weight z-score from 0 to 2 years of age, deviation from midparental height z-score at age 20 and DEXA outcomes [total fat mass (g), total lean mass (g), and soft tissue mass (g)] at age 20, were all assessed using linear regression. Log transformations were performed on skewed outcomes (fat mass, lean mass and soft tissue mass), in which case results were presented as geometric means and percent increase or decrease relative to the reference group (the lowest tertile or undetectable phthalate metabolite levels). Robust standard errors were used where the assumption of heteroscedasticity was violated. Results reported in the text for growth from 0 to 20 years, growth in infancy, and deviation from parental height, were converted to their original units (cm, kg or kg/m^2^) using the CDC growth curves as the reference population, and LMS (lambda-mu-sigma) parameters [38,39]. Differences in height or BMI up to 20 years of age, by phthalate metabolite level, were converted at the average age of the participants within each age group (at 0.56, 5.56, and 14.87 years, during infancy, childhood and adolescence respectively). Age at measurement was adjusted for in the age adjusted analyses, and the linear mixed models were also adjusted for polynomials of age, as its effect on growth z-score was not linear. Where appropriate, multivariately adjusted analyses also included birthweight group for gestational age, midparental height z-score, gestational age at birth, BMI at age eight, maternal prepregnancy BMI group, smoking during pregnancy and age at menarche.

All hypothesis tests were two sided with *p*-values of <0.05 considered statistically significant. In this exploratory, hypothesis-generating study of associations between maternal phthalates and offspring’s outcome data, no adjustments for multiple testing were made, as recommended by the American Statistical Association for such exploratory analyses [42,43]. SAS Enterprise Guide version 7.1 (SAS institute Inc., Cary, NC, USA) and STATA version 16 (StataCorp LLC., College Station, TX, USA) statistical software were used for data analysis.

### 2.6. Ethical Approval

This study was approved by the Raine Study Executive Committee, the Human Research Ethics Committee of Princess Margaret Hospital and the University of Western Australia. Adolescent participants and their accompanying parent or guardian provided written consent.

## 3. Results

The study cohort included 1342 singleton females, 515 of whom had maternal phthalate samples available, and 462 of whom also had at least one outcome measure available. Participant numbers included in each analysis are presented in Figure 1. The Gen2 participants included in this study were slightly older at their 20-year-old follow up and at their DEXA scan than their counterparts not included in the analysis, but were otherwise similar (Table 2).

### 3.1. Age at Menarche

There were no significant associations of menarchal age with maternal phthalate metabolite concentrations in the unadjusted models (Table 3). After adjustment for birthweight for gestational age, gestational age at birth, BMI at eight-year follow-up and maternal smoking during pregnancy, middle tertile mono-(2-carboxymethyl-hexyl) phthalate (MCMHP) (median 12.95 years, inter quartile range [IQR] 12.21–13.81) was associated with later age at menarche (adjusted hazard ratio [aHR] 0.71, 95% CI: 0.52, 0.98, *p* = 0.035), when compared to the lowest tertile of MCMHP (median 12.78 years, IQR 12.02, 13.48). The middle tertile of the sum of the high molecular weight phthalate metabolites (∑high MW phth.metab) (median 13.01 years, IQR 12.18, 13.75) was associated with later age at menarche (aHR 0.72, 95% CI: 0.53, 0.99, *p* = 0.045), when compared to the lowest tertile (median 12.75 years, IQR 12.00, 13.42). Finally, the upper tertile of the sum of the di-iso-nonyl phthalate metabolites (∑DiNPmetab) (median 13.08 years, IQR 12.21, 13.81) was associated with later age at menarche (aHR 0.73, 95% CI: 0.53, 1.00, *p* = 0.048) when compared with the lowest tertile (median 12.83 years, IQR 12.03, 13.50).

### 3.2. Height before Two Years of Age

The average age at follow-up was 1.15 years (range: 1.00–1.78). In the age-adjusted model, a negative association was observed in change of height z-score and maternal phthalate metabolite concentrations for mono-(3-hydroxybutyl) phthalate (MHBP) and mono-carboxy-iso-octyl phthalate (MCiOP) (Supplementary Table S3). After further adjustment for birthweight group, gestational age at birth and midparental height, significant negative associations remained between levels of MHBP and MCIOP and change in height z-scores. The middle tertile of mono-ethyl phthalate (MEP) compared to the lowest tertile was also associated with reduced change in height z-scores (Table 4). Before two years of age, participants with detectable levels of MHBP or MCiOP grew on average 0.73 cm (95% CI: 1.19, 0.26, *p* = 0.002) and 0.66 cm (95% CI: 1.12, 0.20, *p* = 0.005) less than those with undetectable levels, respectively. Participants in the middle tertile of MEP grew 0.57 cm (95% CI: 1.14, 0.01, *p* = 0.047) less than those in the lowest tertile.

### 3.3. Height up to 20 Years of Age

After adjustment for birthweight, gestational age at birth, midparental height and age at menarche, significant positive relationships were detected between height z-score and higher exposure to mono-benzyl phthalate (MBzP) and mono-(2-ethyl-5-carboxypentyl) phthalate (MECPP) (Table 4) between two and 10 years of age. Participants with detectable levels of MBzP were 1.19 cm (95% CI: 0.59, 1.74, *p* = 0.001) taller than participants with undetectable levels of MBzP. Participants in the middle and upper tertiles of MECPP were 0.99 cm (95% CI: 0.30, 1.64, *p* = 0.034) and 0.99 cm (95% CI: 0.30, 1.64, *p* = 0.031) taller than those in the lowest tertile, respectively. Similar associations were observed for MBzP in the age adjusted model (Supplementary Table S3).

### 3.4. Deviation from Midparental Height at 20 Years of Age

There were no age adjusted (Supplementary Table S3) or fully adjusted (Table 4) associations between maternal serum phthalate metabolite level and deviation from midparental height at 20 year follow-up. Height z-scores at age 20 for participants included in this analysis were, on average, 0.26 (95% CI: 0.15, 0.38, *p* < 0.001) higher than their parents, which is equivalent to 1.70 cm (95% CI: 0.57, 2.59).

### 3.5. Weight before Two Years of Age

After adjustment for birthweight group, gestational age at birth and maternal pre-pregnancy BMI, higher levels of MHBP and MCiOP were negatively associated with the change in weight z-score (Table 5). Between birth and their final follow-up before two years of age, participants with detectable levels of MHBP or MCiOP gained on average 0.22 kg (95% CI: 0.41, 0.02, *p* = 0.033) and 0.36 kg (95% CI: 0.55, 0.17, *p* < 0.001) less than those with undetectable levels, respectively. Similar associations for MHBP, MCiOP and MCMHP were observed in the age-adjusted model (Supplementary Table S4).

### 3.6. BMI between Two and Twenty Years of Age

After adjustment for birthweight group, gestational age at birth, maternal prepregnancy BMI and age at menarche, a positive association was observed in adolescence between BMI z-scores and maternal phthalate metabolite concentrations for MHBP, and a negative association for mono-3-carboxypropyl phthalate (MCPP) (Table 5). Participants with detectable levels of MHBP had an average BMI greater by 0.71 kg/m^2^ (95% CI: 0.19, 0.1.3, *p* = 0.033) than those with undetectable levels. Also, participants with detectable levels of MCPP had an average BMI less by 0.79 kg/m^2^ (95% CI: 1.24, 0.27, *p* = 0.033) than those with undetectable levels. Results from the age-adjusted model were similar (Supplementary Table S4).

### 3.7. DEXA Measures of Body Composition at 20 Years of Age

There were no significant associations between fat mass and any maternal serum phthalate metabolite concentration. There was a significant positive age-adjusted association between lean mass and middle tertile mono-(2-ethyl-hexyl) phthalate (MEHP) (Supplementary Table S5). After further adjustment for birthweight group and gestational age at birth, there were significant positive associations between lean mass and MEHP and between soft tissue mass and mono-iso-butyl phthalate (MiBP) (Table 6). However, the relationships between maternal serum phthalate metabolite concentrations and DEXA outcomes were few, and not persistent.

## 4. Discussion

This longitudinal study of the prospective association between maternal phthalate metabolite exposure during pregnancy and age at onset of menarche, as well as childhood and adolescent growth and body composition, demonstrated mixed findings. We observed that higher maternal concentrations of some of the higher molecular weight phthalates, including MCMHP, ∑DiNPmetab and ∑high MW phth.metab, were associated with slightly later age at menarche of about three months on average. No consistent associations were detected between maternal phthalate exposure and offspring BMI, or body composition measures by DEXA. As expected, this study confirmed a generational incremental height increase of Gen2 daughters compared with their parents, which has been observed in many countries [44].

Significant negative associations between prenatal exposure to MEP, MHBP and MCiOP, and infancy growth up to two years of age were found, as well as weak positive associations of participants’ height z-score between two and 10 years of age with exposure to some of the higher molecular weight phthalate metabolites including MBzP and MECPP. This is in contrast to our study in males within the Raine Study [11], where the association was generally found with the lower molecular weight phthalates. Sexually dimorphic influences of prenatal exposure to phthalates on growth parameters have been reported by Valvi et al. who found that prenatal exposure to high molecular weight phthalates was associated with lower BMI z-scores in boys at 4–7 years of age, and a trend towards higher BMI z-scores in girls [45].

Our findings of a negative association of prenatal exposure to several phthalates with growth up to two years of age are consistent with the study of Boas et al. [46], who found that MCiOP urinary phthalate concentrations were negatively associated with thyroid hormones and insulin-like growth factor 1 serum concentrations, as well as with childhood growth. Our findings were not consistent with Botton et al. [32], who despite noting a negative influence of prenatal phthalate exposure on fetal growth, noted a positive association of early childhood growth with some prenatal phthalate exposures (MBzP, MiBP).

The finding of a potential association of delayed menarche with prenatal exposures to phthalate metabolites is consistent with previous reports by Berger et al. [47], who hypothesized, as did we, that the higher molecular weight phthalates would lead to greater obesity and, consequently, earlier puberty in girls. However, this was not what we observed. Despite reports of accelerated Tanner staging due to prenatal phthalate exposure [22], on average, menarche occurred slightly later. Berger et al. speculated that thelarche stage misclassification may occur among the heavier girls, due to the difficulty in differentiating between mammary and adipose tissue, even with manual palpation [47]. We did not record thelarche in our study. With respect to menarche delay, Berger et al. stated that most phthalate metabolites were associated with later menarche, on average between two to three months in normal-weight girls, and they concluded that their results suggest that childhood obesity is an effect modifier, but not a mediator for some of the phthalates [47]. With respect to postnatal exposures on pubertal development, a recent systematic review of adolescent phthalate exposure, which included three studies, derived no evidence of an influence of phthalate exposure on timing of menarche [48].

With respect to BMI, or body composition measurements by DEXA, our results were less consistent across age groups and across different phthalate metabolites. Interpretation of the data can be complicated due to some phthalates having differential effects at different stages of development. This is further compounded by the difficulty of controlling for the known influences of parental BMI, gestational smoking, diet in childhood, socio-economic status, and phthalate exposure in adolescence, which appears to increase abdominal fat deposition [49]. Furthermore, due to the nonlinear dose-response for some phthalates, and the many contributors to growth and adiposity, analysis of prenatal influences is challenging [27]. Potential mechanisms for any associations of prenatal phthalate influences on age at menarche, growth and fat mass accrual through adolescence, may relate to influences on the hypothalamic-pituitary gonadal axis, the growth hormone and/or the thyroid hormone axes. The literature is fairly consistent in showing associations of childhood phthalate exposure being negatively association with concentrations of insulin-like growth factor-1 (IGF-1) and its binding protein (IGF-BP). [46,50]. The literature is not universal in this regard, however, as IGF-1 concentrations rise significantly during puberty, so timing of these measurements during childhood is crucial [50].

In previous studies within the Center for the Health Assessment of Mothers and Children of Salinas (CHAMACOS), urinary metabolites of diethyl phthalate (DEP), butyl benzyl phthalate (BBzP), and di(2-ethylhexyl) phthalate (DEHP) were positively associated with BMI z-score, waist circumference z-score and percent body fat in girls up to 12 years of age [30]. The authors concluded that phthalates were potential obesogens that may explain variability in childhood BMI [25]. Prenatal exposure to the phthalate metabolite MEP was positively associated with BMI level across ages, and prenatal exposure to DEHP was positively associated with increasing and then stabilizing BMI in adolescence [27], similar to Yang et al. [29]. The literature to date of prenatal exposure to phthalates on early life weight gain has recently been summarized by Qian et al. [51]. They concluded that the findings are discrepant, and appear sexually dimorphic, similar to our conclusion. We noted in our male assessment [11], that associations were predominantly positive for prenatal phthalate exposure on BMI through childhood and adolescence, but negative for fat tissue measures, whereas in this study of girls, we detected no consistent findings for BMI trajectory or DEXA measures.

An alternative way to interpret the data derived is that phthalates may be associated with influences on birthweight and gestation at delivery, which subsequently lead to a negative influence of height at two years of age (i.e., prenatal growth and gestation at delivery are mediators of height). Unfortunately, there is limited data available to analyse prenatal growth.

### Limitations

We cannot exclude that our findings are coincidental or even caused by multiple statistical testing. It also would have increased our study power considerably to have urine samples from pregnancy as a matrix for exposure assessment, as phthalate concentrations in urine are higher than in serum, i.e., more samples become detectable. This increases the sensitivity to find linear associations, and contamination problems during sampling are significantly less [4]. It is known that the enzymes involved in hydrolysis of diester phthalates to monoester phthalates are present in blood. They may be responsible for diester to monoester conversion after blood samples are drawn [52]. For that reason, analysis of monoester phthalates may yield falsely elevated levels due to ex vivo conversion of diester contamination during blood sampling, storage and handling in the laboratory. Thus, to inhibit enzyme activity, best practice requires that acid should be added to blood samples immediately after collection and centrifugation for serum isolation. Since samples in the present study were collected 20 years ago, and were stored without acid addition, we performed a series of pilot studies to ensure that the results of the phthalate analyses were robust and likely to reflect true phthalate exposure at time of collection. Diester contamination and conversion after/during the drawing of blood, centrifugation, aliquoting and storage might well have occurred. As we have previously reported [19,20], it is also important to note that the serum levels of phthalate metabolites in the present study are in accordance with levels observed in other studies. For instance, the median levels in serum from U.S. adults (NHANES 1999–2000) were reported to be 4.1 ng/mL (MEP), 14.4 ng/mL (MBP) and 5.4 ng/mL (MEHP) [53]. A Swedish study showed levels in the similar range 11.6 ng/mL (MEP), 13.5 ng/mL (MiBP) and 4.53 ng/mL (MEHP) [54]. In a study of young Danish men, the median MEHP serum concentrations was reported as 7.88 ng/mL [55]. It is entirely possible that exposures occurring now could be less than in the aforementioned studies, with a study comparing samples from historical birth cohorts showing exposure may have decreased between the early 1990s and 2010s (17). It should also be noted that results may have been confounded by participants’ exposure to phthalates and other endocrine disrupting chemicals postnatally.

It is known that obese children potentially have accelerated growth in childhood, consistent with some degree of insulin resistance and peripheral aromatisation of adrenal androgens and, during puberty, gonadal androgens [56]. It is thus possible that our findings of a positive association between height z-score and maternal phthalate metabolite concentrations may be secondary to changes in body fat distribution which are not well captured by BMI z-scores. The associations were predominantly significant during childhood and attenuated in adolescence and at final height. Furthermore, our study design did not offer the opportunity to control for additional postnatal exposures to phthalates, or other endocrine disrupting chemicals, or lifestyle factors that are known to also affect body composition. Additionally, as phthalates are nonpersistent chemicals, exposure can vary from day to day; however, evidence suggests that individuals tend to follow their exposure trajectories [57]. Therefore, we believe that despite the potential collection and storage limitations, the phthalate metabolite levels measured in this study are likely to reflect in vivo exposure at the time of collection. Although it is important to emphasise that any associations derived from these analyses of antenatal phthalate exposure cannot confirm a direct causal relation to outcome.

## 5. Conclusions

Our findings suggest that maternal exposure to the higher molecular weight phthalate metabolites is associated with somewhat later age at menarche. While this finding is statistically significant, its biological or clinical significance is uncertain, and we should not over-interpret the data. No consistent findings were detected for an association between exposure to phthalates and adiposity through childhood, and only weak associations were detected with exposure to some phthalate metabolites for growth in infancy and height between two and 10 years of age.

## Figures and Tables

**Table 1:** Phthalate diesters and their respective metabolites detected in maternal serum during pregnancy, and sums of metabolites used for statistical analysis.

Phthalate Diester	Abbreviation	Human Serum Metabolite	Abbreviation
Di-methyl phthalate	DMP	Mono-methyl phthalate	MMP ^a^
Di-ethyl phthalate	DEP	Mono-ethyl phthalate	MEP
Di-iso-propyl phthalate	DiPrP	Mono-(4-oxopentyl) phthalate	MiPrP ^a^
	DPrP	Mono-propyl phthalate	MPrP ^a^
Di-iso-butyl phthalate	DiBP	Mono-iso-butyl phthalate	MiBP
Di-*n*-butyl phthalate	DnBP	Mono-n-butyl phthalate	MnBP
		Mono-(3-hydroxybutyl) phthalate	MHBP
Butylbenzyl phthalate	BBzP	Mono-benzyl phthalate	MBzP
Di-n-pentyl phthalate	DPP	Mono-n-pentyl phthalate	MPP ^a^
		Mono-(4-hydroxypentyl) phthalate	MHPP ^a^
Di-(2-ethyl-hexyl) phthalate	DEHP	Mono-(2-ethyl-hexyl) phthalate	MEHP
		Mono-(2-ethyl-5-hydroxyhexyl) phthalate	MEHHP ^a^
		Mono- (2-ethyl-5-oxohexyl) phthalate	MEOHP ^a^
		Mono-(2-ethyl-5-carboxypentyl) phthalate	MECPP
		Mono-(2-carboxymethyl-hexyl) phthalate	MCMHP
Di-n-hexyl phthalate	DHxP	Mono-n-hexyl phthalate	MHxP ^a^
		Mono-(5-hydroxyhexyl) phthalate	MHHxP ^a^
		Mono-(5-carboxypentyl) phthalate	MCPeP ^a^
Di-cyclohexyl phthalate	DCHP	Mono-cyclohexyl phthalate	MCHP ^a^
Di-n-heptyl phthalate	DHpP	Mono-n-heptyl phthalate	MhepP ^a^
		Mono-(6-hydroxyheptyl) phthalate	MHHpP ^a^
		Mono-(6-carboxyhexyl) phthalate	MCHxP ^a^
Di-octyl phthalate	DOP	Mono-octyl phthalate	MOP ^a^
		Mono-3-carboxypropyl phthalate	MCPP
Di-iso-nonyl phthalate	DiNP	Mono-iso-nonyl phthalate	MiNP
		Mono-hydroxy-iso-nonyl phthalate	MHiNP ^a^
		Mono-oxo-iso-nonyl phthalate	MOiNP ^a^
		Mono-carboxy-iso-octyl phthalate	MCiOP
Di-iso-decylphthalate	DiDP	Mono-iso-decyl phthalate	MiDP
		Mono-(9-hydroxydecyl) phthalate	MHiDP ^a^
		Mono-(9-oxodecyl) phthalate	MOiDP ^a^
		Mono-(9-carboxynonyl) phthalate	MCiNP ^a^
**Sums of phthalate metabolites**			
ΣMBP(*i* + *n*)		Sum of MiBP and MnBP in ng/mL	
∑DEHPmetab		Molar sum of MEHP, MCMHP, and MECPP expressed as DEHP in ng/mL	
∑DiNPmetab		Molar sum of MiNP and MCIOP expressed as DiNP in ng/mL	
∑DEHP + DiNPmetab		Molar sum of MEHP, MCMHP, MECPP, MiNP, MCiOP expressed as MEHP in ng/mL	
∑low MW phth.metab		Molar sum of MEP, MiBP, MnBP, and MHBP expressed as MEP in ng/mL	
∑high MW phth.metab		Molar sum of MBzP, MEHP, MCMHP, MECPP, MCPP, MiNP, MCiOP, and MiDP expressed as MEHP in ng/mL	
∑all phth.metab		Molar sum of MEP, MiBP, MnBP, MHBP, MBzP, MEHP, MCMHP, MECPP, MCPP, MiNP, MCiOP, and MiDP expressed as MEHP in ng/mL	

^a^ The proportion of samples greater than the limit of detection were not sufficient for inclusion in the analysis.

**Table 2:** Characteristics of the singleton females in the Raine Study who were (*n* = 462) and were not (*n* = 880) included in the present analysis.

	Included in Study		Not Included in Study		
	(Maternal Serum Phthalate Levels and Outcome Data Available) *n* = 462		(Maternal Serum Phthalate Levels or Outcome Data Unavailable) *n* = 880		
	*N*	Median (IQR) or *N*(%)	*N*	Median (IQR) or *N*(%)	*p*-Value ^a^
**Participants’ characteristics at 20 years**					
Age at 20 years follow up (years)	194	20.1 (19.7–20.4)	427	19.9 (19.7–20.2)	0.007
Age at DEXA scan	165	20.1 (19.7–20.4)	387	19.9 (19.7–20.2)	0.011
Height at 20 years follow up (m)	188	1.7 (1.6–1.7)	419	1.7 (1.6–1.7)	0.574
Weight at 20 years follow up (kg)	188	64.0 (56.6–71.8)	419	64.8 (57.2–73.5)	0.630
Age at menarche ^b^	253	12.9 (12.0–13.6)	515	12.7 (12.0–13.4)	0.173
BMI at 20 years follow up (kg/m^2^)	188	22.8 (20.5–26.3)	419	23.1 (21.1–26.3)	0.322
BMI category	188		419		0.806
Underweight		12 (6.4)		21 (5.0)	
Normal		118 (62.8)		256 (61.1)	
Overweight		32 (17.0)		82 (19.6)	
Obese		26 (13.8)		60 (14.3)	
**Adiposity (DEXA) at 20 yrs**					
Soft tissue percentage	165	41 (34–48)	387	40 (35–47)	0.895
Total fat percentage	165	39 (33–46)	387	39 (33–45)	0.837
Total fat mass (kg)	165	24.4 (18.1–32.2)	387	24.8 (19.4–32.4)	0.521
Total lean mass (kg)	165	36.0 (32.7–39.6)	387	36.7 (33.6–40)	0.245
Total soft tissue mass (kg)	165	61.2 (54.8–67.4)	387	62.1 (55.1–69.9)	0.421
**Maternal characteristics in pregnancy**					
Maternal age at delivery	462	28.1 (23.9–32.1)	875	28 (23.3–32.2)	0.788
Smoked during pregnancy	462	131 (28.3)	875	245 (28.0)	0.891
Drunk alcohol during first trimester	462	202 (43.7)	875	395 (45.1)	0.619
Maternal height (m)	456	1.6 (1.6–1.7)	870	1.6 (1.6–1.7)	0.246
Maternal BMI (pre-pregnancy)	456	21.2 (19.6–23.8)	870	21.3 (19.7–23.6)	0.642
Maternal BMI category	456		870		0.515
Underweight		64 (14.0)		98 (11.3)	
Normal		307 (67.3)		612 (70.3)	
Overweight		54 (11.8)		101 (11.6)	
Obese		31 (6.8)		59 (6.8)	
Maternal education since leaving school	462		880		0.821
Missing		6 (1.3)		10 (1.1)	
None		230 (49.8)		446 (50.7)	
Trade		181 (39.2)		332 (37.7)	
University		45 (9.7)		92 (10.5)	
Maternal Race	462		880		0.055
Caucasian		403 (87.2)		763 (86.7)	
Chinese		30 (6.5)		35 (4.0)	
Indian		10 (2.2)		33 (3.8)	
Other		19 (4.1)		49 (5.6)	
Marital Status	462		880		0.699
Married or de facto		384 (83.1)		724 (82.3)	
Not married or de facto		78 (16.9)		156 (17.7)	
**Participants characteristics at birth**					
Gestational age at birth	462		880		0.188
<34 weeks		13 (2.8)		20 (2.3)	
34–36 weeks		33 (7.1)		43 (4.9)	
37 + weeks		416 (90.0)		817 (92.8)	
Birthweight (kg)	462	3.3 (2.9–3.6)	880	3.3 (3.0–3.6)	0.308
Adjusted birthweight percentile	462		880		0.999
≤3rd		14 (3.0)		26 (3.0)	
4th–10th		37 (8.0)		70 (8.0)	
11th–49th		169 (36.6)		321 (36.5)	
50th–89th		189 (40.9)		360 (40.9)	
≥90th		53 (11.5)		103 (11.7)	
Expected birthweight ratio	462	1.0 (0.9–1.1)	878	1.0 (0.9–1.1)	0.768

^a^ Mann-Whitney tests were used to compare continuous outcomes, and chi-square tests for categorical outcomes. ^b^ Exact date or month of menarche was available for 255 singleton girls with phthalates data and 515 without. Two girls with phthalates were excluded from the analysis. One was an outlier and one had inconsistently coded data.

**Table 3:** Unadjusted and fully adjusted associations between categorized phthalate metabolite levels and age at menarche.

			Unadjusted	Adjusted	
		Median (IQR)	HR (95% CI)	HR (95% CI)	
MEP					
≤1.15		12.96 (12.10, 13.70)	ref	ref	
>1.15 and ≤4.71		12.78 (12.00, 13.50)	1.21 (0.89, 1.65)	1.20 (0.88, 1.64)	
>4.71		12.98 (12.14, 13.74)	0.97 (0.72, 1.31)	0.95 (0.70, 1.29)	
MiBP					
Not Detectable		12.83 (12.02, 13.52)	ref	ref	
Detectable		12.96 (12.10, 13.74)	0.83 (0.64, 1.06)	0.82 (0.63, 1.05)	
MnBP					
≤1.41		12.90 (12.04, 13.68)	ref	ref	
>1.41 and ≤3.44		12.85 (12.03, 13.59)	1.07 (0.79, 1.46)	1.16 (0.84, 1.59)	
>3.44		12.96 (12.08, 13.74)	0.93 (0.69, 1.26)	0.92 (0.68, 1.26)	
MHBP					
Not Detectable		12.89 (12.04, 13.62)	ref	ref	
Detectable		12.95 (12.08, 13.70)	0.93 (0.72, 1.20)	0.91 (0.70, 1.18)	
MBzP					
Not Detectable		12.96 (12.10, 13.74)	ref	ref	
Detectable		12.84 (12.03, 13.53)	1.20 (0.93, 1.53)	1.19 (0.92, 1.53)	
MEHP					
≤2.71		12.85 (12.03, 13.59)	ref	ref	
>2.71 and ≤4.69		12.90 (12.04, 13.68)	0.93 (0.68, 1.26)	0.92 (0.67, 1.25)	
>4.69		12.96 (12.10, 13.71)	0.88 (0.65, 1.19)	0.82 (0.60, 1.12)	
MECPP					
≤0.59		12.84 (12.03, 13.55)	ref	ref	
>0.59 and ≤1.02		13.07 (12.19, 13.82)	0.78 (0.58, 1.05)	0.78 (0.57, 1.07)	
>1.02		12.83 (12.02, 13.52)	1.03 (0.76, 1.41)	0.99 (0.72, 1.36)	
MCMHP					
≤1.03		12.79 (12.02, 13.52)	ref	ref	
>1.03 and ≤1.69		13.05 (12.19, 13.82)	0.74 (0.55, 1.01)	0.71 (0.52, 0.98)	**↑**
>1.69		12.89 (12.04, 13.62)	0.91 (0.67, 1.23)	0.86 (0.64, 1.17)	
MCPP					
Not Detectable		12.92 (12.07, 13.68)	ref	ref	
Detectable		12.89 (12.04, 13.62)	1.05 (0.82, 1.35)	1.20 (0.92, 1.57)	
MiNP					
≤2.77		12.85 (12.04, 13.60)	ref	ref	
>2.77 and ≤4.87		12.92 (12.07, 13.68)	0.93 (0.68, 1.25)	0.95 (0.69, 1.30)	
>4.87		12.95 (12.08, 13.70)	0.91 (0.67, 1.24)	0.82 (0.60, 1.12)	
MCiOP					
Not Detectable		12.85 (12.03, 13.59)	ref	ref	
Detectable		12.96 (12.10, 13.74)	0.86 (0.67, 1.11)	0.83 (0.64, 1.07)	
MiDP					
Not Detectable		12.89 (12.04, 13.64)	ref	ref	
Detectable		12.92 (12.07, 13.68)	0.98 (0.76, 1.26)	1.01 (0.78, 1.31)	
∑MBP(i + *n*)					
≤2.06		12.84 (12.03, 13.53)	ref	ref	
>2.06 and ≤5.14		12.89 (12.04, 13.62)	0.94 (0.69, 1.27)	0.93 (0.68, 1.26)	
>5.14		12.99 (12.15, 13.77)	0.82 (0.60, 1.11)	0.80 (0.59, 1.09)	
∑DEHPmetab					
≤6.59		12.96 (12.10, 13.74)	ref	ref	
>6.59 and ≤9.73		12.78 (12.01, 13.50)	1.24 (0.91, 1.68)	1.11 (0.81, 1.52)	
>9.73		12.96 (12.10, 13.74)	1.00 (0.74, 1.35)	0.93 (0.69, 1.26)	
∑DiNPmetab					
≤4.15		12.84 (12.03, 13.55)	ref	ref	
>4.15 and ≤7.31		12.90 (12.04, 13.68)	0.92 (0.68, 1.25)	0.92 (0.67, 1.27)	
>7.31		12.98 (12.14, 13.75)	0.84 (0.62, 1.14)	0.73 (0.53, 1.00)	**↑**
∑DEHP + DiNPmetab					
≤8.42		12.87 (12.04, 13.62)	ref	ref	
>8.42 and ≤11.72		12.87 (12.04, 13.62)	0.99 (0.73, 1.34)	0.92 (0.68, 1.25)	
>11.72		12.98 (12.14, 13.75)	0.86 (0.63, 1.18)	0.77 (0.56, 1.07)	
∑low MW phth.metab					
≤4.2		12.90 (12.04, 13.68)	ref	ref	
>4.2 and ≤10.46		12.76 (12.01, 13.50)	1.16 (0.85, 1.58)	1.16 (0.85, 1.58)	
>10.46		13.04 (12.16, 13.79)	0.86 (0.64, 1.17)	0.84 (0.62, 1.14)	
∑high MW phth.metab					
≤9.22		12.76 (12.01, 13.48)	ref	ref	
>9.22 and ≤13.43		12.96 (12.10, 13.74)	0.79 (0.58, 1.07)	0.72 (0.53, 0.99)	**↑**
>13.43		12.96 (12.10, 13.74)	0.80 (0.58, 1.09)	0.76 (0.55, 1.04)	
∑all phth.metab					
≤17.39		12.89 (12.04, 13.62)	ref	ref	
>17.39 and ≤28.0		12.84 (12.03, 13.52)	1.07 (0.79, 1.44)	1.05 (0.77, 1.42)	
>28.0		13.04 (12.18, 13.79)	0.83 (0.61, 1.13)	0.79 (0.58, 1.08)	

Adjusted analyses were adjusted for birthweight for gestational age, gestational age at birth, BMI at eight-year follow-up and maternal smoking during pregnancy. **↑** Indicates that the menarche occurred at a later age with higher maternal serum phthalate levels. Data for 369 girls were included in this analysis. Exact date or month of menarche was available for 253 girls. Where no menarche data were available (*n* = 104), age at menarche was censored at the later available age of the eighth or 10-year follow up. If only year of menarche was available (*n* = 12), age at menarche was censored at the age at the start of the year.

**Table 4:** Summary of adjusted associations between maternal serum phthalate metabolite levels and deviation from mid-parental height at 20 years of age; height z-scores between 0 and 20 years of age, and change in height z-score between 0 and two years of age.

	Change in Height from 0–2 Years of Age (*N* = 335) Change Z-Score		Linear Mixed Model for Heights ^a^ (*N* = 270) Marginal Mean Z-Score (95% CI)						Deviation from Mid-Parental Height at 20 Years of Age (*N* = 165) Deviation Z-Score	
	β_Phthalate_ (95% CI)		0–2 Years		2–10 Years		10–20 Years		β_Phthalate_ (95% CI)	
MEP										
≤1.15	ref		−0.12 (−0.28, 0.04)		0.00 (−0.14, 0.14)		0.60 (0.43, 0.77)		ref	
>1.15 and ≤4.71	−0.19 (−0.38, 0.00)	**↓**	−0.20 (−0.37, −0.04)		−0.02 (−0.16, 0.12)		0.59 (0.42, 0.76)		−0.02 (−0.30, 0.26)	
>4.71	0.00 (−0.19, 0.18)		−0.19 (−0.35, −0.03)		0.13 (−0.01, 0.26)		0.70 (0.53, 0.87)		0.02 (−0.26, 0.31)	
MiBP										
Not Detectable	ref		−0.23 (−0.38, −0.08)		−0.03 (−0.15, 0.10)		0.56 (0.41, 0.71)		ref	
Detectable	−0.07 (−0.23, 0.09)		−0.13 (−0.27, 0.02)		0.09 (−0.03, 0.20)		0.68 (0.54, 0.81)		0.17 (−0.08, 0.42)	
MnBP		^b^								
≤1.41	ref		−0.17 (−0.33, 0.00)		−0.02 (−0.16, 0.12)		0.60 (0.44, 0.77)		ref	
>1.41 and ≤3.44	0.12 (−0.07, 0.30)		−0.08 (−0.24, 0.08)		0.09 (−0.04, 0.23)		0.64 (0.47, 0.81)		0.14 (−0.14, 0.42)	
>3.44	−0.15 (−0.34, 0.04)		−0.26 (−0.42, −0.10)		0.03 (−0.11, 0.17)		0.63 (0.46, 0.80)		0.20 (−0.10, 0.49)	
MHBP										
Not Detectable	ref		−0.12 (−0.26, 0.02)		0.05 (−0.06, 0.16)		0.64 (0.50, 0.77)		ref	
Detectable	−0.24 (−0.40, −0.09)	**↓**	−0.24 (−0.40, −0.09)		0.01 (−0.11, 0.14)		0.61 (0.46, 0.77)		0.07 (−0.16, 0.30)	
MBzP										
Not Detectable	ref		−0.18 (−0.33, −0.04)		−0.08 (−0.19, 0.04)		0.55 (0.41, 0.69)		ref	
Detectable	−0.04 (−0.20, 0.12)		−0.14 (−0.29, 0.01)		0.16 (0.04, 0.27)	**↑**	0.71 (0.56, 0.85)		0.15 (−0.09, 0.39)	
MEHP										
≤2.71	ref		−0.15 (−0.32, 0.01)		0.12 (−0.02, 0.26)		0.69 (0.52, 0.86)		ref	
>2.71 and ≤4.69	0.04 (−0.15, 0.23)		−0.12 (−0.28, 0.04)		−0.02 (−0.16, 0.12)		0.57 (0.40, 0.74)		0.14 (−0.15, 0.42)	
>4.69	−0.01 (−0.20, 0.18)		−0.25 (−0.41, −0.08)		0.02 (−0.12, 0.16)		0.62 (0.45, 0.79)		0.06 (−0.23, 0.36)	
MECPP										
≤0.59	ref		−0.20 (−0.37, −0.04)		−0.10 (−0.24, 0.04)		0.51 (0.34, 0.68)		ref	
>0.59 and ≤1.02	0.06 (−0.14, 0.25)		−0.10 (−0.26, 0.06)		0.10 (−0.04, 0.23)	**↑**	0.70 (0.53, 0.87)		0.21 (−0.08, 0.49)	
>1.02	−0.10 (−0.29, 0.09)		−0.19 (−0.35, −0.03)		0.10 (−0.04, 0.23)	**↑**	0.66 (0.49, 0.83)		−0.02 (−0.30, 0.26)	
MCMHP										
≤1.03	ref		−0.19 (−0.35, −0.02)		0.06 (−0.07, 0.20)		0.59 (0.42, 0.76)		ref	
>1.03 and ≤1.69	−0.04 (−0.23, 0.15)		−0.20 (−0.37, −0.03)		0.01 (−0.14, 0.15)		0.63 (0.46, 0.81)		0.05 (−0.23, 0.33)	
>1.69	0.00 (−0.19, 0.18)		−0.14 (−0.30, 0.02)		0.04 (−0.09, 0.18)		0.66 (0.49, 0.82)		−0.09 (−0.37, 0.18)	
MCPP										
Not Detectable	ref		−0.14 (−0.28, 0.00)		0.08 (−0.03, 0.20)		0.66 (0.52, 0.79)		ref	
Detectable	−0.04 (−0.20, 0.12)		−0.22 (−0.37, −0.06)		−0.03 (−0.15, 0.10)		0.59 (0.43, 0.74)		0.14 (−0.09, 0.37)	
MiNP										
≤2.77	ref		−0.20 (−0.36, −0.03)		−0.03 (−0.17, 0.12)		0.63 (0.46, 0.81)		ref	
>2.77 and ≤4.87	−0.11 (−0.31, 0.08)		−0.14 (−0.30, 0.03)		0.00 (−0.14, 0.13)		0.59 (0.43, 0.76)		−0.19 (−0.46, 0.09)	
>4.87	−0.10 (−0.3, 0.10)		−0.16 (−0.32, 0.00)		0.12 (−0.01, 0.25)		0.65 (0.48, 0.82)		−0.16 (−0.46, 0.15)	
MCiOP										
Not Detectable	ref		−0.10 (−0.25, 0.04)		0.05 (−0.07, 0.18)		0.64 (0.49, 0.78)		ref	
Detectable	−0.22 (−0.38, −0.07)	**↓**	−0.23 (−0.38, −0.09)		0.02 (−0.10, 0.14)		0.62 (0.48, 0.76)		0.04 (−0.19, 0.27)	
MiDP										
Not Detectable	ref		−0.20 (−0.34, −0.07)		0.09 (−0.02, 0.20)		0.66 (0.53, 0.79)		ref	
Detectable	0.07 (−0.09, 0.23)		−0.11 (−0.26, 0.05)		−0.05 (−0.18, 0.08)		0.57 (0.42, 0.73)		0.05 (−0.18, 0.29)	
∑MBP(i + *n*)										
≤2.06	ref		−0.17 (−0.33, 0.00)		−0.04 (−0.18, 0.10)		0.56 (0.39, 0.74)		ref	
>2.06 and ≤5.14	−0.07 (−0.26, 0.13)		−0.12 (−0.28, 0.04)		0.06 (−0.07, 0.20)		0.65 (0.49, 0.82)		−0.03 (−0.31, 0.25)	
>5.14	−0.18 (−0.38, 0.01)		−0.22 (−0.38, −0.06)		0.07 (−0.07, 0.21)		0.66 (0.49, 0.83)		0.19 (−0.11, 0.48)	
∑DEHPmetab										
≤6.59	ref		−0.20 (−0.36, −0.04)		−0.04 (−0.18, 0.10)		0.55 (0.37, 0.72)		ref	
>6.59 and ≤9.73	0.03 (−0.16, 0.23)		−0.10 (−0.26, 0.06)		0.12 (−0.02, 0.26)		0.69 (0.51, 0.86)		0.07 (−0.22, 0.35)	
>9.73	−0.10 (−0.29, 0.09)		−0.22 (−0.38, −0.06)		0.03 (−0.11, 0.17)		0.65 (0.48, 0.82)		0.09 (−0.18, 0.37)	
∑DiNPmetab										
≤4.15	ref		−0.19 (−0.35, −0.02)		−0.03 (−0.18, 0.11)		0.61 (0.44, 0.79)		ref	
>4.15 and ≤7.31	−0.08 (−0.27, 0.12)		−0.13 (−0.29, 0.04)		0.00 (−0.14, 0.14)		0.63 (0.46, 0.80)		−0.08 (−0.36, 0.19)	
>7.31	−0.12 (−0.32, 0.07)		−0.18 (−0.34, −0.02)		0.12 (−0.02, 0.25)		0.63 (0.47, 0.80)		−0.12 (−0.42, 0.18)	
∑DEHP + DiNPmetab										
≤8.42	ref		−0.24 (−0.41, −0.07)		−0.04 (−0.19, 0.10)		0.59 (0.41, 0.76)		ref	
>8.42 and ≤11.72	0.05 (−0.14, 0.24)		−0.07 (−0.23, 0.09)		0.13 (0.00, 0.26)		0.69 (0.53, 0.85)		0.08 (−0.20, 0.36)	
>11.72	−0.11 (−0.30, 0.08)		−0.22 (−0.38, −0.06)		0.01 (−0.13, 0.15)		0.60 (0.43, 0.77)		−0.07 (−0.35, 0.21)	
∑low MW phth.metab										
≤4.2	ref		−0.12 (−0.28, 0.04)		0.01 (−0.13, 0.15)		0.64 (0.47, 0.81)		ref	
>4.2 and ≤10.46	−0.12 (−0.31, 0.08)		−0.16 (−0.32, 0.00)		0.01 (−0.13, 0.15)		0.57 (0.4, 0.74)		0.01 (−0.28, 0.31)	
>10.46	−0.10 (−0.29, 0.09)		−0.23 (−0.39, −0.07)		0.09 (−0.05, 0.23)		0.67 (0.50, 0.84)		0.00 (−0.28, 0.29)	
∑high MW phth.metab										
≤9.22	ref		−0.23 (−0.40, −0.06)		0.01 (−0.13, 0.15)		0.60 (0.42, 0.77)		ref	
>9.22 and ≤13.43	0.01 (−0.18, 0.20)		−0.09 (−0.25, 0.07)		0.08 (−0.06, 0.21)		0.68 (0.51, 0.84)		0.18 (−0.10, 0.45)	
>13.43	−0.04 (−0.23, 0.16)		−0.20 (−0.36, −0.04)		0.02 (−0.12, 0.16)		0.60 (0.43, 0.77)		−0.01 (−0.29, 0.26)	
∑all phth.metab										
≤17.39	ref		−0.12 (−0.28, 0.05)		0.04 (−0.10, 0.18)		0.64 (0.47, 0.81)		ref	
>17.39 and ≤28.0	−0.05 (−0.24, 0.14)		−0.15 (−0.31, 0.01)		−0.02 (−0.16, 0.11)		0.63 (0.46, 0.80)		0.04 (−0.24, 0.31)	
>28.0	−0.16 (−0.35, 0.02)		−0.24 (−0.41, −0.08)		0.09 (−0.04, 0.23)		0.61 (0.44, 0.78)		0.01 (−0.27, 0.30)	

^a^ Results for linear mixed models are presented as marginal means (95% CI), while results for other analyses are presented as beta coefficients (95% CI). The analyses of deviation from midparental height were adjusted for age at 20 year follow up, birthweight for gestational age and mid-parental height. The linear mixed models were adjusted for age at measurement, birthweight for gestational age, mid parental height, gestational age at birth and age at menarche. The analyses of growth from 0–2 years were adjusted for length at birth, age at follow up, birthweight for gestational age, midparental height and gestational age at birth. **↑** Indicates that the outcome increased with higher maternal serum phthalate levels. **↓** Indicates that the outcome decreased with higher maternal serum phthalate levels. ^b^ Indicates a significant overall effect of the maternal serum phthalate levels on the outcome.

**Table 5:** Summary of adjusted associations between maternal serum phthalate metabolite levels and BMI z-scores between two and 20 years of age and change in weight z-score between 0 and two years of age.

	Change in Weight from 0–2 Years of Age (*N* = 409) Change Z-Score		Linear Mixed Model for BMI ^a^ (*N* = 319) Marginal Mean Z-Score (95% CI)			
	β_Phthalate_ (95% CI)		2–10 Years		10–20 Years	
MEP						
≤1.15	ref		0.15 (−0.03, 0.33)		0.18 (0.00, 0.35)	
>1.15 and ≤4.71	−0.07 (−0.30, 0.17)		0.37 (0.20, 0.53)		0.27 (0.10, 0.44)	
>4.71	0.09 (−0.14, 0.33)		0.28 (0.10, 0.45)		0.22 (0.05, 0.40)	
MiBP						
Not Detectable	ref		0.26 (0.11, 0.41)		0.22 (0.07, 0.38)	
Detectable	−0.03 (−0.22, 0.16)		0.28 (0.14, 0.41)		0.22 (0.09, 0.36)	
MnBP						
≤1.41	ref		0.27 (0.09, 0.44)		0.19 (0.02, 0.37)	
>1.41 and ≤3.44	−0.01 (−0.25, 0.23)		0.29 (0.11, 0.46)		0.23 (0.05, 0.40)	
>3.44	−0.15 (−0.38, 0.09)		0.26 (0.09, 0.43)		0.25 (0.08, 0.42)	
MHBP						
Not Detectable	ref		0.19 (0.06, 0.32)		0.14 (0.01, 0.27)	
Detectable	−0.21 (−0.40, −0.02)	**↓**	0.39 (0.23, 0.54)		0.35 (0.20, 0.51)	**↑**
MBzP						
Not Detectable	ref		0.27 (0.14, 0.41)		0.22 (0.08, 0.36)	
Detectable	−0.04 (−0.23, 0.15)		0.26 (0.12, 0.41)		0.22 (0.08, 0.37)	
MEHP						
≤2.71	ref		0.30 (0.12, 0.48)		0.25 (0.07, 0.43)	
>2.71 and ≤4.69	−0.16 (−0.39, 0.07)		0.19 (0.02, 0.36)		0.20 (0.02, 0.37)	
>4.69	0.02 (−0.21, 0.25)		0.32 (0.15, 0.49)		0.23 (0.06, 0.40)	
MECPP						
≤0.59	ref		0.30 (0.13, 0.48)		0.19 (0.01, 0.36)	
>0.59 and ≤1.02	−0.06 (−0.29, 0.18)		0.25 (0.08, 0.42)		0.24 (0.06, 0.41)	
>1.02	−0.15 (−0.38, 0.09)		0.26 (0.08, 0.43)		0.25 (0.07, 0.43)	
MCMHP						
≤1.03	ref		0.34 (0.17, 0.51)		0.27 (0.10, 0.44)	
>1.03 and ≤1.69	−0.20 (−0.44, 0.03)		0.17 (−0.01, 0.34)		0.16 (−0.02, 0.33)	
>1.69	−0.04 (−0.26, 0.19)		0.30 (0.13, 0.47)		0.24 (0.07, 0.41)	
MCPP						
Not Detectable	ref		0.34 (0.21, 0.46)		0.32 (0.19, 0.45)	
Detectable	−0.06 (−0.25, 0.13)		0.17 (0.02, 0.33)		0.08 (−0.07, 0.24)	**↓**
MiNP						
≤2.77	ref		0.21 (0.03, 0.38)		0.14 (−0.03, 0.32)	
>2.77 and ≤4.87	0.11 (−0.13, 0.35)		0.37 (0.19, 0.54)		0.32 (0.14, 0.49)	
>4.87	0.00 (−0.24, 0.24)		0.24 (0.07, 0.41)		0.21 (0.04, 0.38)	
MCiOP						
Not Detectable	ref		0.35 (0.2, 0.49)		0.28 (0.13, 0.43)	
Detectable	−0.35 (−0.54, −0.16)	**↓**	0.20 (0.06, 0.34)		0.17 (0.04, 0.31)	
MiDP						
Not Detectable	ref		0.27 (0.14, 0.40)		0.26 (0.13, 0.39)	
Detectable	−0.04 (−0.23, 0.16)		0.27 (0.12, 0.43)		0.17 (0.01, 0.33)	
∑MBP(i + *n*)						
≤2.06	ref		0.25 (0.07, 0.43)		0.14 (−0.04, 0.32)	
>2.06 and ≤5.14	−0.20 (−0.44, 0.04)		0.29 (0.13, 0.46)		0.27 (0.10, 0.44)	
>5.14	−0.18 (−0.41, 0.06)		0.26 (0.09, 0.43)		0.25 (0.08, 0.43)	
∑DEHPmetab						
≤6.59	ref		0.26 (0.08, 0.43)		0.18 (0.01, 0.36)	
>6.59 and ≤9.73	−0.14 (−0.38, 0.09)		0.22 (0.05, 0.39)		0.21 (0.03, 0.38)	
>9.73	−0.04 (−0.27, 0.19)		0.32 (0.16, 0.49)		0.28 (0.11, 0.45)	
∑DiNPmetab						
≤4.15	ref		0.22 (0.05, 0.39)		0.17 (−0.01, 0.34)	
>4.15 and ≤7.31	0.10 (−0.13, 0.34)		0.32 (0.14, 0.49)		0.31 (0.13, 0.49)	
>7.31	−0.05 (−0.29, 0.19)		0.27 (0.10, 0.44)		0.20 (0.03, 0.37)	
∑DEHP + DiNPmetab						
≤8.42	ref		0.16 (−0.01, 0.34)		0.17 (−0.01, 0.35)	
>8.42 and ≤11.72	0.06 (−0.17, 0.30)		0.36 (0.19, 0.52)		0.28 (0.11, 0.45)	
>11.72	−0.09 (−0.32, 0.15)		0.27 (0.10, 0.44)		0.21 (0.04, 0.38)	
∑low MW phth.metab						
≤4.2	ref		0.18 (0.00, 0.35)		0.14 (−0.04, 0.31)	
>4.2 and ≤10.46	−0.15 (−0.39, 0.08)		0.33 (0.16, 0.50)		0.30 (0.13, 0.47)	
>10.46	0.00 (−0.23, 0.24)		0.30 (0.12, 0.47)		0.23 (0.06, 0.40)	
∑high MW phth.metab						
≤9.22	ref		0.20 (0.02, 0.38)		0.17 (−0.01, 0.35)	
>9.22 and ≤13.43	0.00 (−0.24, 0.23)		0.30 (0.14, 0.47)		0.25 (0.07, 0.42)	
>13.43	−0.02 (−0.26, 0.21)		0.30 (0.13, 0.47)		0.25 (0.08, 0.42)	
∑all phth.metab						
≤17.39	ref		0.18 (0.01, 0.36)		0.13 (−0.05, 0.31)	
>17.39 and ≤28.0	−0.08 (−0.32, 0.16)		0.26 (0.09, 0.42)		0.26 (0.09, 0.43)	
>>28.0	−0.15 (−0.39, 0.08)		0.36 (0.19, 0.53)		0.27 (0.10, 0.45)	

^a^ Results for linear mixed models are presented as marginal means (95% CI), while results for change in z-score from 0–2 years are presented as beta coefficients (95% CI). The linear mixed models were adjusted for age at measurement, birthweight for gestational age, maternal pre-pregnancy BMI, gestational age at birth and age at menarche. The analyses of growth from 0–2 years were adjusted for weight at birth, age at follow up, birthweight for gestational age, maternal prepregnancy BMI and gestational age at birth. **↑** Indicates that the outcome increased with higher maternal serum phthalate levels. **↓** Indicates that the outcome decreased with higher maternal serum phthalate levels.

**Table 6:** Adjusted associations between categorized phthalate metabolite levels and DEXA outcomes (*N* = 165).

	Fat Mass (g)		Lean Mass (g)		Soft Tissue Mass (g)	
	Geometric Mean (95% CI)		Geometric Mean (95% CI)		Geometric Mean (95% CI)	
MEP						
≤1.15	ref		ref		ref	
>1.15 and ≤4.71	0.91 (0.78, 1.06)		0.99 (0.94, 1.05)		0.96 (0.88, 1.04)	
>4.71	0.93 (0.80, 1.09)		0.98 (0.92, 1.03)		0.96 (0.89, 1.04)	
MiBP						
Not Detectable	ref		ref		ref	
Detectable	1.11 (0.97, 1.27)		1.03 (0.98, 1.08)		1.07 (1.00, 1.14)	**↑**
MnBP						
≤1.41	ref		ref		ref	
>1.41 and ≤3.44	1.00 (0.86, 1.16)		1.03 (0.97, 1.08)		1.02 (0.95, 1.09)	
>3.44	1.05 (0.89, 1.23)		1.05 (0.99, 1.11)		1.05 (0.97, 1.14)	
MHBP						
Not Detectable	ref		ref		ref	
Detectable	1.05 (0.93, 1.19)		1.04 (0.99, 1.09)		1.05 (0.98, 1.11)	
MBzP						
Not Detectable	ref		ref		ref	
Detectable	0.96 (0.84, 1.09)		1.00 (0.96, 1.05)		0.98 (0.92, 1.05)	
MEHP				**^a^**		
≤2.71	ref		ref		ref	
>2.71 and ≤4.69	1.02 (0.86, 1.19)		1.08 (1.02, 1.14)	**↑**	1.05 (0.98, 1.14)	
>4.69	0.93 (0.78, 1.09)		1.03 (0.97, 1.09)		0.98 (0.91, 1.06)	
MECPP^¥^						
≤0.59	ref		ref		ref	
>0.59 and ≤1.02	0.89 (0.76, 1.05)		1.02 (0.96, 1.08)		0.97 (0.89, 1.05)	
>1.02	0.94 (0.80, 1.11)		1.01 (0.96, 1.07)		0.98 (0.91, 1.07)	
MCMHP						
≤1.03	ref		ref		ref	
>1.03 and ≤1.69	0.89 (0.76, 1.03)		0.99 (0.94, 1.05)		0.95 (0.88, 1.02)	
>1.69	0.94 (0.81, 1.09)		1.03 (0.97, 1.08)		0.99 (0.92, 1.07)	
MCPP						
Not Detectable	ref		ref		ref	
Detectable	0.95 (0.84, 1.08)		0.99 (0.95, 1.04)		0.98 (0.92, 1.04)	
MiNP						
≤2.77	ref		ref		ref	
>2.77 and ≤4.87	1.07 (0.92, 1.23)		0.96 (0.91, 1.02)		1.00 (0.93, 1.07)	
>4.87	0.94 (0.79, 1.11)		0.97 (0.91, 1.03)		0.96 (0.88, 1.04)	
MCiOP						
Not Detectable	ref		ref		ref	
Detectable	0.96 (0.85, 1.09)		1.02 (0.98, 1.07)		1.00 (0.94, 1.06)	
MiDP						
Not Detectable	ref		ref		ref	
Detectable	1.02 (0.90, 1.16)		0.97 (0.93, 1.02)		0.99 (0.93, 1.06)	
∑MBP(i + *n*)						
≤2.06	ref		ref		ref	
>2.06 and ≤5.14	1.08 (0.93, 1.26)		0.99 (0.94, 1.05)		1.03 (0.96, 1.11)	
>5.14	1.08 (0.92, 1.27)		1.05 (0.99, 1.11)		1.07 (0.99, 1.16)	
∑DEHPmetab						
≤6.59	ref		ref		ref	
>6.59 and ≤9.73	0.91 (0.77, 1.06)		1.00 (0.95, 1.06)		0.96 (0.89, 1.04)	
>9.73	0.93 (0.80, 1.08)		1.02 (0.96, 1.07)		0.98 (0.91, 1.05)	
∑DiNPmetab						
≤4.15	ref		ref		ref	
>4.15 and ≤7.31	1.06 (0.92, 1.23)		0.97 (0.92, 1.02)		1.00 (0.93, 1.07)	
>7.31	0.91 (0.77, 1.08)		0.97 (0.91, 1.03)		0.95 (0.87, 1.03)	
∑DEHP + DiNPmetab						
≤8.42	ref		ref		ref	
>8.42 and ≤11.72	1.02 (0.88, 1.19)		1.00 (0.95, 1.06)		1.01 (0.94, 1.09)	
>11.72	0.92 (0.79, 1.07)		0.99 (0.94, 1.05)		0.96 (0.89, 1.03)	
∑low MW phth.metab						
≤4.2	ref		ref		ref	
>4.2 and ≤0.46	1.14 (0.98, 1.33)		1.00 (0.94, 1.05)		1.05 (0.97, 1.13)	
>10.46	1.04 (0.89, 1.22)		1.01 (0.95, 1.07)		1.03 (0.95, 1.11)	
∑high MW phth.metab						
≤9.22	ref		ref		ref	
>9.22 and ≤13.43	1.01 (0.86, 1.17)		1.04 (0.98, 1.10)		1.02 (0.95, 1.10)	
>13.43	0.96 (0.83, 1.12)		1.00 (0.94, 1.05)		0.98 (0.91, 1.06)	
∑all phth.metab						
≤17.39	ref		ref		ref	
>17.39 and ≤28.0	1.01 (0.87, 1.17)		1.03 (0.97, 1.08)		1.02 (0.95, 1.10)	
>28.0	1.02 (0.87, 1.19)		1.02 (0.96, 1.08)		1.02 (0.95, 1.11)	

Fat mass, lean mass and soft tissue mass were log transformed and results are presented as geometric means. Analyses were adjusted for age at DEXA scan, birthweight for gestational age and gestational age at birth. **↑** Indicates that the outcome increased with higher maternal serum phthalate levels. **^a^** Indicates a significant overall effect of the maternal serum phthalate levels on the outcome.

## Data Availability

The data underlying this article were provided by The Raine Study by permission. Data will be shared on request to the corresponding author with permission of The Raine Study.

## Supplementary Materials

The following are available online at https://www.mdpi.com/article/10.3390/ijerph18094725/s1. Supplementary Table S1: Number of participants with BMI and height data included in the linear mixed models, at each follow-up. Supplementary Table S2: Phthalate metabolites (ng/mL) detected in maternal serum during pregnancy for female participants who had phthalates and any outcome measures available (N = 462), or were included in a sub-analysis. Supplementary Table S3: Summary of age adjusted associations between maternal serum phthalate metabolite levels and deviation from mid-parental height at 20 years of age; height z-scores between 0 and 20 years of age and change in height z-score between 0 and two years of age. Supplementary Table S4: Summary of age adjusted associations between maternal serum phthalate metabolite levels and BMI z-scores between two and 20 years of age and change in weight z-score between 0 and two years of age. Supplementary Table S5: Age-adjusted associations between categorized phthalate metabolite levels and DEXA outcome.
