# Supplementary material for: Associations between Prenatal Exposure to Phthalates and Timing of Menarche and Growth and Adiposity into Adulthood: A Twenty-Years Birth Cohort Study
Source: Int J Environ Res Public Health. 2021 Apr 29;18(9):4725. doi: 10.3390/ijerph18094725 (PMC8125681; doi:10.3390/ijerph18094725)
Supplement: Supplementary file 1 [file ijerph-18-04725-s001.zip › ijerph-1172642-supplementary.pdf]

Supplementary Table 1. Number of participants with BMI and height data included in the linear mixed models, at each follow-up.

	Number of participants with data available	
**Follow-up age**	**BMI (N=319)**	**Height (N=270)**
birth	n/a	269
1	n/a	253
2	88	80
3	228	194
5	279	237
8	280	242
10	269	231
14	227	197
17	169	147
20	178	159

Supplementary Table 2. Phthalate metabolites (ng/mL) detected in maternal serum during pregnancy for female participants who had phthalates and any outcome measures available (N = 462), or were included in a sub-analysis.

		Phthalates and any outcome *N=462*				Linear mixed model for heights *N=270*				Linear mixed model for BMI *N=319*			
	LOD (ng/mL)	% > LOD	Min	Median	Max	% > LOD	Min	Median	Max	% > LOD	Min	Median	Max
MEP	0.65	75.1	<LOD	2.6	2106.5	76.3	<LOD	2.5	284.2	76.2	<LOD	2.6	284.2
MiBP	0.75	57.4	<LOD	1.1	74.0	57.4	<LOD	1.0	16.1	57.1	<LOD	1.1	16.1
MnBP	0.61	84.4	<LOD	2.2	463.5	85.6	<LOD	2.3	386.1	85.6	<LOD	2.4	386.1
MHBP	0.22	41.3	<LOD	<LOD	2.5	39.3	<LOD	<LOD	1.0	40.8	<LOD	<LOD	1.9
MBzP	0.26	46.8	<LOD	<LOD	16.4	46.7	<LOD	<LOD	5.6	46.4	<LOD	<LOD	8.1
MEHP	0.74	100.0	1.2	3.4	23.1	100.0	1.2	3.4	22.5	100.0	1.2	3.6	22.5
MECPP	0.25	87.9	<LOD	0.8	7.4	85.6	<LOD	0.8	7.4	86.8	<LOD	0.8	7.4
MCMHP	0.39	97.4	<LOD	1.3	36.5	96.7	<LOD	1.3	36.5	96.9	<LOD	1.3	36.5
MCPP	0.19	40.0	<LOD	<LOD	5.5	42.2	<LOD	<LOD	4.1	40.8	<LOD	<LOD	4.1
MINP	0.53	94.6	<LOD	3.9	12.5	95.6	<LOD	4.2	12.5	95.3	<LOD	4.1	12.5
MCiOP	0.13	51.5	<LOD	0.1	2.7	52.2	<LOD	0.1	2.7	53.3	<LOD	0.1	2.7
MiDP	0.72	37.9	<LOD	<LOD	21.2	40.7	<LOD	<LOD	21.2	40.4	<LOD	<LOD	21.2
∑MBP(i+n)		100.0	<LOD	3.3	532.8	100.0	<LOD	3.3	402.3	100.0	<LOD	3.4	402.3
∑DEHPmetab		100.0	1.8	8.0	64.6	100.0	1.8	8.1	64.6	100.0	1.8	8.3	64.6
∑DiNPmetab		100.0	<LOD	6.0	18.1	100.0	<LOD	6.3	18.1	100.0	<LOD	6.3	18.1
∑DEHP+DiNPmetab		100.0	1.3	9.9	46.9	100.0	1.3	10.0	46.9	100.0	1.3	10.1	46.9
∑low MW phth.metab		100.0	<LOD	6.5	2572.5	100.0	<LOD	6.2	444.8	100.0	<LOD	6.4	444.8
∑high MW phth.metab		100.0	1.3	11.1	46.9	100.0	1.3	11.3	46.9	100.0	1.3	11.3	46.9
∑all phth.metab		100.0	1.3	21.6	3738.0	100.0	1.3	21.4	676.0	100.0	1.3	21.7	676.0

		Deviation from mid-parental height at 20 years *N=165*				Linear model for DEXA scan outcomes at age 20 *N=165*				Cox Regression for Age at Menarche *N=369*			
	LOD (ng/mL)	% > LOD	Min	Median	Max	% > LOD	Min	Median	Max	% > LOD	Min	Median	Max
MEP	0.65	75.8	<LOD	2.4	92.8	74.6	<LOD	2.4	92.8	74.8	<LOD	2.6	2106.5
MiBP	0.75	55.2	<LOD	0.9	16.1	52.1	<LOD	0.9	16.1	56.1	<LOD	1.0	69.3
MnBP	0.61	84.2	<LOD	2.4	386.1	81.8	<LOD	2.3	386.1	84.0	<LOD	2.1	463.5
MHBP	0.22	38.8	<LOD	<LOD	1.0	37.0	<LOD	<LOD	1.9	40.7	<LOD	<LOD	2.0
MBzP	0.26	46.7	<LOD	<LOD	5.6	45.5	<LOD	<LOD	8.1	47.2	<LOD	<LOD	16.4
MEHP	0.74	100.0	1.2	3.7	22.5	100.0	1.2	3.9	22.5	100.0	1.2	3.4	23.1
MECPP	0.25	86.1	<LOD	0.8	7.2	84.9	<LOD	0.8	7.2	86.5	<LOD	0.8	7.4

MCMHP	0.39	95.8	<LOD	1.3	18.1	95.2	<LOD	1.4	18.1	97.0	<LOD	1.4	36.5
MCPP	0.19	39.4	<LOD	<LOD	4.1	39.4	<LOD	<LOD	2.5	40.4	<LOD	<LOD	4.1
MINP	0.53	94.6	<LOD	4.0	10.4	93.9	<LOD	3.8	10.4	94.3	<LOD	4.0	12.5
MCiOP	0.13	48.5	<LOD	<LOD	2.7	48.5	<LOD	0.1	2.7	52.0	<LOD	0.1	2.7
MiDP	0.72	37.0	<LOD	<LOD	12.0	39.4	<LOD	<LOD	12.0	38.5	<LOD	<LOD	21.2
∑MBP(i+n)		100.0	<LOD	3.4	402.3	100.0	<LOD	3.3	402.3	100.0	<LOD	3.3	532.8
∑DEHPmetab		100.0	1.8	8.8	44.3	100.0	1.8	8.9	44.3	100.0	1.8	8.0	64.6
∑DiNPmetab		100.0	<LOD	6.1	15.5	100.0	<LOD	5.7	15.5	100.0	<LOD	6.2	18.1
∑DEHP+DiNPmetab		100.0	1.3	10.0	33.4	100.0	1.3	10.0	33.4	100.0	1.3	10.0	46.9
∑low MW phth.metab		100.0	<LOD	6.3	444.8	100.0	<LOD	5.9	444.8	100.0	<LOD	6.3	2572.5
∑high MW phth.metab		100.0	1.3	11.4	34.7	100.0	1.3	11.5	34.7	100.0	1.3	11.0	46.9
∑all phth.metab		100.0	1.3	22.3	676.0	100.0	1.3	21.0	676.0	100.0	1.3	21.1	3738.0

		0-2 HEIGHT *N=335*				0-2 BMI *N=409*			
	LOD (ng/mL)	% > LOD	Min	Median	Max	% > LOD	Min	Median	Max
MEP	0.65	75.5	<LOD	2.6	2106.5	76.0	<LOD	2.8	2106.5
MiBP	0.75	58.8	<LOD	1.1	74.0	58.2	<LOD	1.1	74.0
MnBP	0.61	86.3	<LOD	2.3	463.5	85.6	<LOD	2.3	463.5
MHBP	0.22	41.8	<LOD	<LOD	2.5	42.3	<LOD	<LOD	2.5
MBzP	0.26	47.5	<LOD	<LOD	16.4	47.7	<LOD	<LOD	16.4
MEHP	0.74	100.0	1.2	3.4	23.1	100.0	1.2	3.4	23.1
MECPP	0.25	87.5	<LOD	0.9	7.4	88.5	<LOD	0.8	7.4
MCMHP	0.39	97.3	<LOD	1.4	36.5	97.6	<LOD	1.4	36.5
MCPP	0.19	40.3	<LOD	<LOD	5.5	39.6	<LOD	<LOD	5.5
MINP	0.53	95.8	<LOD	4.2	12.5	95.1	<LOD	4.1	12.5
MCiOP	0.13	53.7	<LOD	0.1	2.7	52.6	<LOD	0.1	2.7
MiDP	0.72	36.4	<LOD	<LOD	21.2	37.2	<LOD	<LOD	21.2
∑MBP(i+n)		100.0	<LOD	3.4	532.8	100.0	<LOD	3.4	532.8
∑DEHPmetab		100.0	1.8	8.3	64.6	100.0	1.8	8.3	64.6
∑DiNPmetab		100.0	<LOD	6.3	18.1	100.0	<LOD	6.3	18.1
∑DEHP+DiNPmetab		100.0	1.3	10.1	46.9	100.0	1.3	10.1	46.9
∑low MW phth.metab		100.0	<LOD	6.8	2572.5	100.0	<LOD	6.8	2572.5
∑high MW phth.metab		100.0	1.3	11.3	46.9	100.0	1.3	11.3	46.9
∑all phth.metab		100.0	1.3	23.0	3738.0	100.0	1.3	22.4	3738.0

Supplementary Table 3. Summary of age adjusted associations between maternal serum phthalate metabolite levels and deviation from mid-parental height at 20 years of age; height z-scores between 0 and 20 years of age and change in height z-score between 0 and 2 years of age

	Change in height from 0-2 years of age (N=335) Change z-score	Linear mixed model for heights[a] (N=270) Marginal Mean z-score (95% CI)			Deviation from mid-parental height at 20 years of age (N=165) Deviation z-score
	$\beta_{Phthalate}$ (95% CI)	0-2 years	2-10 years	10-20 years	$\beta_{Phthalate}$ (95% CI)
MEP					
≤1.15	ref	-0.17 (-0.35, 0.02)	0.06 (-0.11, 0.23)	0.52 (0.32, 0.72)	ref
>1.15 and ≤4.71	-0.07 (-0.30, 0.17)	-0.19 (-0.38, 0.00)	0.11 (-0.06, 0.27)	0.58 (0.38, 0.78)	-0.01 (-0.30, 0.28)
>4.71	-0.04 (-0.28, 0.19)	-0.28 (-0.46, -0.09)	0.16 (-0.01, 0.32)	0.62 (0.42, 0.82)	-0.03 (-0.31, 0.26)
MiBP					
Not Detectable	ref	-0.27 (-0.44, -0.09)	0.05 (-0.10, 0.20)	0.52 (0.34, 0.69)	ref
Detectable	-0.11 (-0.31, 0.09)	-0.17 (-0.33, -0.01)	0.15 (0.02, 0.29)	0.61 (0.46, 0.77)	0.14 (-0.12, 0.40)
MnBP					
≤1.41	ref	-0.15 (-0.34, 0.03)	0.11 (-0.06, 0.28)	0.59 (0.39, 0.78)	ref
>1.41 and ≤3.44	-0.02 (-0.25, 0.22)	-0.18 (-0.37, 0.01)	0.11 (-0.05, 0.28)	0.54 (0.35, 0.74)	0.12 (-0.17, 0.41)
>3.44	-0.21 (-0.44, 0.03)	-0.29 (-0.48, -0.11)	0.11 (-0.06, 0.27)	0.59 (0.39, 0.79)	0.18 (-0.12, 0.48)
MHBP					
Not Detectable	ref	-0.15 (-0.31, 0.01)	0.14 (0.01, 0.27)	0.59 (0.43, 0.74)	ref
Detectable	-0.31 (-0.50, -0.11) ↓	-0.30 (-0.48, -0.13)	0.07 (-0.09, 0.22)	0.55 (0.37, 0.73)	0.09 (-0.15, 0.33)
MBzP					
Not Detectable	ref	-0.24 (-0.40, -0.08)	-0.01 (-0.15, 0.13)	0.48 (0.32, 0.64)	ref
Detectable	0.00 (-0.19, 0.20)	-0.16 (-0.33, 0.00)	0.24 (0.10, 0.39) ↑	0.68 (0.51, 0.85)	0.11 (-0.14, 0.36)
MEHP					
≤2.71	ref	-0.15 (-0.34, 0.04)	0.23 (0.07, 0.40)	0.69 (0.49, 0.89)	ref
>2.71 and ≤4.69	-0.06 (-0.30, 0.17)	-0.19 (-0.37, -0.01)	0.02 (-0.15, 0.18)	0.48 (0.28, 0.67)	0.13 (-0.16, 0.43)
>4.69	-0.04 (-0.27, 0.20)	-0.29 (-0.48, -0.11)	0.09 (-0.08, 0.25)	0.56 (0.36, 0.75)	0.03 (-0.27, 0.34)
MECPP[¥]					
≤0.59	ref	-0.22 (-0.41, -0.03)	0.01 (-0.17, 0.18)	0.49 (0.29, 0.69)	ref

>0.59 and ≤1.02	0.01 (-0.23, 0.25)	-0.15 (-0.34, 0.04)	0.16 (-0.01, 0.33)	0.65 (0.45, 0.84)	0.13 (-0.17, 0.42)
>1.02	-0.10 (-0.34, 0.13)	-0.24 (-0.43, -0.06)	0.15 (-0.01, 0.32)	0.58 (0.38, 0.77)	-0.06 (-0.35, 0.24)
MCMHP					
≤1.03	ref	-0.25 (-0.43, -0.06)	0.12 (-0.05, 0.28)	0.52 (0.32, 0.72)	ref
>1.03 and ≤1.69	-0.13 (-0.37, 0.10)	-0.22 (-0.42, -0.03)	0.10 (-0.08, 0.27)	0.60 (0.39, 0.8)	0.05 (-0.24, 0.33)
>1.69	-0.07 (-0.30, 0.16)	-0.17 (-0.36, 0.01)	0.12 (-0.04, 0.28)	0.60 (0.41, 0.79)	-0.14 (-0.42, 0.14)
MCPP					
Not Detectable	ref	-0.16 (-0.32, -0.01)	0.17 (0.04, 0.31)	0.62 (0.47, 0.78)	ref
Detectable	-0.02 (-0.22, 0.17)	-0.28 (-0.45, -0.11)	0.03 (-0.12, 0.18)	0.51 (0.33, 0.69)	0.08 (-0.15, 0.32)
MiNP					
≤2.77	ref	-0.23 (-0.42, -0.04)	0.07 (-0.11, 0.24)	0.58 (0.38, 0.78)	ref
>2.77 and ≤4.87	-0.09 (-0.33, 0.15)	-0.16 (-0.34, 0.03)	0.09 (-0.07, 0.26)	0.57 (0.37, 0.77)	-0.19 (-0.47, 0.09)
>4.87	-0.08 (-0.32, 0.17)	-0.24 (-0.42, -0.05)	0.16 (-0.01, 0.32)	0.57 (0.37, 0.76)	-0.16 (-0.47, 0.15)
MCiOP					
Not Detectable	ref	-0.14 (-0.31, 0.02)	0.13 (-0.01, 0.27)	0.59 (0.42, 0.76)	ref
Detectable	-0.21 (-0.40, -0.02) ↓	-0.27 (-0.44, -0.11)	0.09 (-0.04, 0.23)	0.56 (0.40, 0.72)	0.05 (-0.19, 0.28)
MiDP					
Not Detectable	ref	-0.22 (-0.38, -0.06)	0.18 (0.05, 0.31)	0.63 (0.48, 0.79)	ref
Detectable	-0.02 (-0.22, 0.18)	-0.18 (-0.36, -0.01)	0.00 (-0.15, 0.15)	0.48 (0.29, 0.66)	0.08 (-0.16, 0.32)
∑MBP(i+n)					
≤2.06	ref	-0.16 (-0.35, 0.03)	0.09 (-0.08, 0.26)	0.56 (0.35, 0.76)	ref
>2.06 and ≤5.14	-0.08 (-0.32, 0.16)	-0.22 (-0.40, -0.03)	0.09 (-0.07, 0.25)	0.55 (0.36, 0.74)	-0.06 (-0.34, 0.23)
>5.14	-0.17 (-0.41, 0.07)	-0.25 (-0.44, -0.06)	0.15 (-0.02, 0.32)	0.61 (0.41, 0.81)	0.16 (-0.14, 0.46)
∑DEHPmetab					
≤6.59	ref	-0.19 (-0.38, 0.00)	0.08 (-0.09, 0.25)	0.54 (0.34, 0.74)	ref
>6.59 and ≤9.73	-0.05 (-0.28, 0.19)	-0.18 (-0.37, 0.00)	0.15 (-0.02, 0.31)	0.59 (0.39, 0.79)	0.03 (-0.26, 0.32)
>9.73	-0.15 (-0.38, 0.09)	-0.26 (-0.44, -0.07)	0.10 (-0.07, 0.27)	0.59 (0.39, 0.78)	0.07 (-0.22, 0.35)
∑DiNPmetab					
≤4.15	ref	-0.21 (-0.40, -0.02)	0.07 (-0.10, 0.24)	0.57 (0.37, 0.77)	ref
>4.15 and ≤7.31	-0.03 (-0.27, 0.21)	-0.15 (-0.34, 0.03)	0.09 (-0.07, 0.26)	0.60 (0.40, 0.80)	-0.1 (-0.38, 0.18)

>7.31	-0.09 (-0.33, 0.15)	-0.25 (-0.43, -0.07)	0.15 (-0.01, 0.31)	0.55 (0.36, 0.74)	-0.14 (-0.45, 0.18)
∑DEHP+DiNPmetab					
≤8.42	ref	-0.26 (-0.45, -0.07)	0.05 (-0.12, 0.23)	0.56 (0.35, 0.76)	ref
>8.42 and ≤11.72	0.07 (-0.16, 0.31)	-0.11 (-0.29, 0.07)	0.20 (0.04, 0.36)	0.63 (0.44, 0.82)	0.17 (-0.11, 0.45)
>11.72	-0.08 (-0.32, 0.16)	-0.28 (-0.46, -0.09)	0.07 (-0.10, 0.23)	0.52 (0.32, 0.72)	-0.05 (-0.34, 0.24)
∑low MW phth.metab					
≤4.2	ref	-0.16 (-0.35, 0.03)	0.08 (-0.09, 0.25)	0.57 (0.37, 0.77)	ref
>4.2 and ≤10.46	-0.10 (-0.34, 0.14)	-0.24 (-0.43, -0.05)	0.04 (-0.13, 0.21)	0.49 (0.29, 0.68)	-0.02 (-0.32, 0.28)
>10.46	-0.07 (-0.31, 0.16)	-0.23 (-0.41, -0.04)	0.20 (0.04, 0.37)	0.66 (0.46, 0.85)	-0.01 (-0.31, 0.28)
∑high MW phth.metab					
≤9.22	ref	-0.24 (-0.43, -0.05)	0.11 (-0.06, 0.28)	0.58 (0.37, 0.78)	ref
>9.22 and ≤13.43	0.00 (-0.24, 0.24)	-0.12 (-0.30, 0.06)	0.16 (0.00, 0.33)	0.64 (0.45, 0.84)	0.21 (-0.08, 0.5)
>13.43	-0.05 (-0.29, 0.19)	-0.28 (-0.46, -0.09)	0.06 (-0.11, 0.22)	0.50 (0.31, 0.70)	-0.01 (-0.29, 0.28)
∑all phth.metab					
≤17.39	ref	-0.11 (-0.30, 0.08)	0.16 (-0.01, 0.33)	0.63 (0.43, 0.83)	ref
>17.39 and ≤28.0	0.04 (-0.20, 0.28)	-0.20 (-0.39, -0.02)	0.04 (-0.13, 0.20)	0.57 (0.37, 0.76)	0.03 (-0.26, 0.32)
>28.0	-0.19 (-0.43, 0.05)	-0.31 (-0.50, -0.13)	0.14 (-0.03, 0.30)	0.52 (0.32, 0.72)	-0.02 (-0.32, 0.27)

[a]results for linear mixed models are presented as marginal means (95% CI), while results for other analyses are presented as beta coefficients (95% CI)

-deviation from mid-parental height analysis adjusted for age at 20 year follow up and mid-parental height

-linear mixed model adjusted for age at measurement

-growth from 0-2 years analysis adjusted for length at birth and age at follow up

-↑ Indicates that the outcome increased with higher maternal serum phthalate levels.

-↓ Indicates that the outcome decreased with higher maternal serum phthalate levels.

Supplementary Table 4. Summary of age adjusted associations between maternal serum phthalate metabolite levels and BMI z-scores between 2 and 20 years of age and change in weight z-score between 0 and 2 years of age

	Change in weight from 0-2 years of age (N=409) change z-score	Linear mixed model for BMI[a] (N=319) Marginal Mean z-score (95% CI)	
	$\beta_{Phthalate}$ (95% CI)	2-10 years	10-20 years
MEP			
≤1.15	ref	0.16 (-0.02, 0.35)	0.19 (0.01, 0.38)
>1.15 and ≤4.71	-0.02 (-0.27, 0.22)	0.38 (0.20, 0.55)	0.28 (0.11, 0.46)
>4.71	0.08 (-0.16, 0.33)	0.25 (0.07, 0.43)	0.21 (0.02, 0.39)
MiBP			
Not Detectable	ref	0.26 (0.10, 0.42)	0.23 (0.07, 0.39)
Detectable	-0.03 (-0.24, 0.17)	0.28 (0.14, 0.41)	0.23 (0.09, 0.37)
MnBP			
≤1.41	ref	0.27 (0.09, 0.45)	0.2 (0.02, 0.39)
>1.41 and ≤3.44	-0.02 (-0.27, 0.23)	0.26 (0.08, 0.44)	0.21 (0.03, 0.39)
>3.44	-0.16 (-0.41, 0.09)	0.27 (0.09, 0.45)	0.27 (0.09, 0.45)
MHBP			
Not Detectable	ref	0.18 (0.05, 0.32)	0.13 (0, 0.27)
Detectable	-0.23 (-0.43, -0.02) ↓	0.39 (0.23, 0.55)	0.37 (0.2, 0.53) ↑
MBzP			
Not Detectable	ref	0.26 (0.12, 0.41)	0.22 (0.07, 0.36)
Detectable	0.02 (-0.18, 0.22)	0.27 (0.12, 0.43)	0.24 (0.09, 0.4)
MEHP			
≤2.71	ref	0.33 (0.14, 0.51)	0.28 (0.09, 0.47)
>2.71 and ≤4.69	-0.18 (-0.42, 0.07)	0.18 (0.00, 0.36)	0.2 (0.02, 0.38)
>4.69	0.04 (-0.21, 0.28)	0.30 (0.13, 0.48)	0.21 (0.04, 0.39)
MECPP[¥]			
≤0.59	ref	0.32 (0.14, 0.50)	0.21 (0.03, 0.39)
>0.59 and ≤1.02	-0.09 (-0.33, 0.16)	0.27 (0.09, 0.44)	0.26 (0.08, 0.44)

>1.02	-0.15 (-0.40, 0.10)		0.22 (0.04, 0.40)	0.22 (0.03, 0.4)	
MCMHP					
≤1.03	ref		0.36 (0.18, 0.54)	0.31 (0.12, 0.49)	
>1.03 and ≤1.69	-0.28 (-0.53, -0.04)	↓	0.19 (0.00, 0.37)	0.18 (0.00, 0.37)	
>1.69	-0.09 (-0.33, 0.15)		0.25 (0.08, 0.43)	0.20 (0.02, 0.38)	
MCPP					
Not Detectable	ref		0.34 (0.21, 0.48)	0.33 (0.19, 0.47)	
Detectable	-0.04 (-0.25, 0.16)		0.16 (0.00, 0.32)	0.08 (-0.08, 0.24)	↓
MiNP					
≤2.77	ref		0.18 (0.00, 0.36)	0.12 (-0.06, 0.31)	
>2.77 and ≤4.87	0.12 (-0.13, 0.36)		0.37 (0.19, 0.55)	0.33 (0.14, 0.51)	
>4.87	0.06 (-0.20, 0.31)		0.26 (0.08, 0.43)	0.24 (0.06, 0.41)	
MCiOP					
Not Detectable	ref		0.33 (0.17, 0.48)	0.27 (0.11, 0.42)	
Detectable	-0.31 (-0.51, -0.11)	↓	0.22 (0.07, 0.36)	0.20 (0.05, 0.34)	
MiDP					
Not Detectable	ref		0.27 (0.13, 0.40)	0.26 (0.13, 0.40)	
Detectable	-0.09 (-0.30, 0.12)		0.27 (0.11, 0.44)	0.18 (0.01, 0.34)	
∑MBP(i+n)					
≤2.06	ref		0.25 (0.06, 0.44)	0.14 (-0.04, 0.33)	
>2.06 and ≤5.14	-0.16 (-0.41, 0.09)		0.28 (0.10, 0.45)	0.26 (0.08, 0.43)	
>5.14	-0.20 (-0.45, 0.05)		0.27 (0.09, 0.46)	0.28 (0.09, 0.46)	
∑DEHPmetab					
≤6.59	ref		0.29 (0.10, 0.47)	0.22 (0.03, 0.40)	
>6.59 and ≤9.73	-0.14 (-0.38, 0.11)		0.21 (0.03, 0.39)	0.20 (0.01, 0.38)	
>9.73	-0.04 (-0.29, 0.20)		0.31 (0.13, 0.49)	0.27 (0.09, 0.45)	
∑DiNPmetab					
≤4.15	ref		0.20 (0.02, 0.38)	0.15 (-0.03, 0.34)	
>4.15 and ≤7.31	0.14 (-0.11, 0.39)		0.31 (0.13, 0.50)	0.31 (0.13, 0.50)	
>7.31	0.02 (-0.23, 0.28)		0.29 (0.11, 0.47)	0.22 (0.05, 0.40)	

∑DEHP+DiNPmetab			
≤8.42	ref	0.14 (-0.05, 0.33)	0.15 (-0.03, 0.34)
>8.42 and ≤11.72	0.11 (-0.13, 0.36)	0.37 (0.19, 0.54)	0.30 (0.12, 0.47)
>11.72	-0.03 (-0.28, 0.22)	0.28 (0.10, 0.46)	0.23 (0.05, 0.41)
∑low MW phth.metab			
≤4.2	ref	0.21 (0.03, 0.39)	0.17 (-0.01, 0.36)
>4.2 and ≤10.46	-0.16 (-0.41, 0.09)	0.28 (0.11, 0.46)	0.26 (0.08, 0.44)
>10.46	-0.02 (-0.27, 0.22)	0.31 (0.13, 0.49)	0.25 (0.07, 0.43)
∑high MW phth.metab			
≤9.22	ref	0.20 (0.01, 0.38)	0.17 (-0.01, 0.36)
>9.22 and ≤13.43	0.01 (-0.23, 0.26)	0.33 (0.15, 0.51)	0.28 (0.09, 0.46)
>13.43	0.00 (-0.25, 0.25)	0.28 (0.10, 0.45)	0.23 (0.06, 0.41)
∑all phth.metab			
≤17.39	ref	0.23 (0.04, 0.41)	0.18 (0.00, 0.37)
>17.39 and ≤28.0	-0.07 (-0.32, 0.18)	0.22 (0.04, 0.39)	0.23 (0.05, 0.40)
>28.0	-0.17 (-0.42, 0.08)	0.36 (0.18, 0.54)	0.28 (0.10, 0.46)

[a]results for linear mixed models are presented as marginal means (95% CI), while results for change in z-score from 0-2 years are presented as beta coefficients (95% CI)

-linear mixed model adjusted for age at measurement,

-growth from 0-2 years analysis adjusted for weight at birth and age at follow up

-↑ Indicates that the outcome increased with higher maternal serum phthalate levels.

-↓ Indicates that the outcome decreased with higher maternal serum phthalate levels.

Supplementary Table 5. Age adjusted associations between categorized phthalate metabolite levels and DEXA outcomes (N=165)

	Fat Mass (g)	Lean Mass (g)	Soft Tissue (g)
	Geometric Mean (95% CI)	Geometric Mean (95% CI)	Geometric Mean (95% CI)
MEP			
≤1.15	ref	ref	ref
>1.15 and ≤4.71	0.92 (0.79, 1.07)	1.00 (0.94, 1.05)	0.96 (0.89, 1.04)
>4.71	0.92 (0.79, 1.07)	0.97 (0.92, 1.02)	0.95 (0.88, 1.02)
MiBP			
Not Detectable	ref	ref	ref
Detectable	1.08 (0.95, 1.24)	1.02 (0.97, 1.07)	1.05 (0.98, 1.12)
MnBP			
≤1.41	ref	ref	ref
>1.41 and ≤3.44	0.99 (0.85, 1.16)	1.03 (0.97, 1.08)	1.01 (0.94, 1.09)
>3.44	1.06 (0.90, 1.24)	1.05 (0.98, 1.12)	1.06 (0.97, 1.14)
MHBP			
Not Detectable	ref	ref	ref
Detectable	1.06 (0.93, 1.20)	1.04 (1.00, 1.09)	1.05 (0.99, 1.12)
MBzP			
Not Detectable	ref	ref	ref
Detectable	0.95 (0.83, 1.09)	1.00 (0.95, 1.05)	0.98 (0.91, 1.05)
MEHP		[a]	
≤2.71	ref	ref	ref
>2.71 and ≤4.69	1.01 (0.86, 1.20)	1.07 (1.01, 1.14) ↑	1.05 (0.96, 1.14)
>4.69	0.92 (0.79, 1.08)	1.01 (0.96, 1.08)	0.97 (0.90, 1.05)
MECPP[¥]			
≤0.59	ref	ref	ref
>0.59 and ≤1.02	0.88 (0.75, 1.02)	1.01 (0.95, 1.07)	0.95 (0.88, 1.02)
>1.02	0.94 (0.80, 1.09)	1.00 (0.95, 1.06)	0.98 (0.90, 1.05)
MCMHP			
≤1.03	ref	ref	ref
>1.03 and ≤1.69	0.88 (0.76, 1.02)	0.99 (0.94, 1.05)	0.94 (0.88, 1.01)
>1.69	0.92 (0.79, 1.06)	1.02 (0.96, 1.08)	0.98 (0.90, 1.06)
MCPP			

Not Detectable	ref	ref	ref
Detectable	0.94 (0.83, 1.07)	0.99 (0.95, 1.04)	0.97 (0.92, 1.04)
MiNP			
≤2.77	ref	ref	ref
>2.77 and ≤4.87	1.06 (0.92, 1.23)	0.96 (0.91, 1.01)	1.00 (0.93, 1.07)
>4.87	0.95 (0.80, 1.12)	0.96 (0.9, 1.03)	0.96 (0.88, 1.04)
MCiOP			
Not Detectable	ref	ref	ref
Detectable	0.97 (0.85, 1.10)	1.02 (0.98, 1.07)	1.00 (0.94, 1.06)
MiDP			
Not Detectable	ref	ref	ref
Detectable	1.03 (0.91, 1.17)	0.97 (0.93, 1.02)	1.00 (0.94, 1.06)
∑MBP(i+n)			
≤2.06	ref	ref	ref
>2.06 and ≤5.14	1.06 (0.91, 1.23)	0.99 (0.94, 1.04)	1.02 (0.95, 1.10)
>5.14	1.08 (0.92, 1.27)	1.05 (0.99, 1.11)	1.07 (0.99, 1.16)
∑DEHPmetab			
≤6.59	ref	ref	ref
>6.59 and ≤9.73	0.90 (0.77, 1.05)	0.99 (0.93, 1.04)	0.95 (0.88, 1.02)
>9.73	0.92 (0.80, 1.07)	1.01 (0.95, 1.06)	0.97 (0.90, 1.05)
∑DiNPmetab			
≤4.15	ref	ref	ref
>4.15 and ≤7.31	1.06 (0.92, 1.22)	0.96 (0.91, 1.01)	0.99 (0.93, 1.07)
>7.31	0.92 (0.78, 1.08)	0.96 (0.91, 1.02)	0.94 (0.87, 1.03)
∑DEHP+DiNPmetab			
≤8.42	ref	ref	ref
>8.42 and ≤11.72	1.05 (0.90, 1.22)	1.01 (0.96, 1.07)	1.02 (0.95, 1.10)
>11.72	0.93 (0.80, 1.08)	0.99 (0.93, 1.04)	0.96 (0.89, 1.03)
∑low MW phth.metab			
≤4.2	ref	ref	ref
>4.2 and ≤10.46	1.12 (0.96, 1.31)	0.99 (0.94, 1.05)	1.04 (0.96, 1.12)
>10.46	1.04 (0.89, 1.22)	1.00 (0.95, 1.06)	1.02 (0.95, 1.10)
∑high MW phth.metab			
≤9.22	ref	ref	ref
>9.22 and ≤13.43	1.01 (0.86, 1.18)	1.04 (0.99, 1.10)	1.02 (0.95, 1.11)
>13.43	0.96 (0.83, 1.12)	0.99 (0.94, 1.05)	0.98 (0.91, 1.05)

∑all phth.metab			
≤17.39	ref	ref	ref
>17.39 and ≤28.0	1.00 (0.86, 1.17)	1.02 (0.97, 1.08)	1.02 (0.94, 1.09)
>28.0	1.01 (0.87, 1.18)	1.01 (0.95, 1.07)	1.01 (0.94, 1.09)

-fat mass, lean mass and soft tissue mass were log transformed and results are presented as geometric means

-↑ Indicates that the outcome increased with higher maternal serum phthalate levels.

-↓ Indicates that the outcome decreased with higher maternal serum phthalate levels.

-[a]Indicates a significant overall effect of the maternal serum phthalate levels on the outcome.
